# Flexible Morphological Regulation of Photothermal Nanodrugs: Understanding the Relationship between the Structure, Photothermal Effect, and Tumoral Biodistribution

**DOI:** 10.1021/acsnano.4c15587

**Published:** 2025-01-10

**Authors:** Shukun Li, Yudong Li, Guizhi Shen, Juping Sun, Loai K. E. A. Abdelmohsen, Xuehai Yan, Jan C. M. van Hest

**Affiliations:** † State Key Laboratory of Biochemical Engineering, Institute of Process Engineering, Beijing 100190, China; § School of Chemical Engineering, University of Chinese Academy of Sciences, Beijing 100049, China; ∥ Bio-Organic Chemistry, Institute for Complex Molecular Systems, Eindhoven University of Technology, P.O. Box 513, 5600 MB Eindhoven, The Netherlands

**Keywords:** morphology regulation, nanodrug, polymer, photothermal effect, biodistribution, antitumor therapy

## Abstract

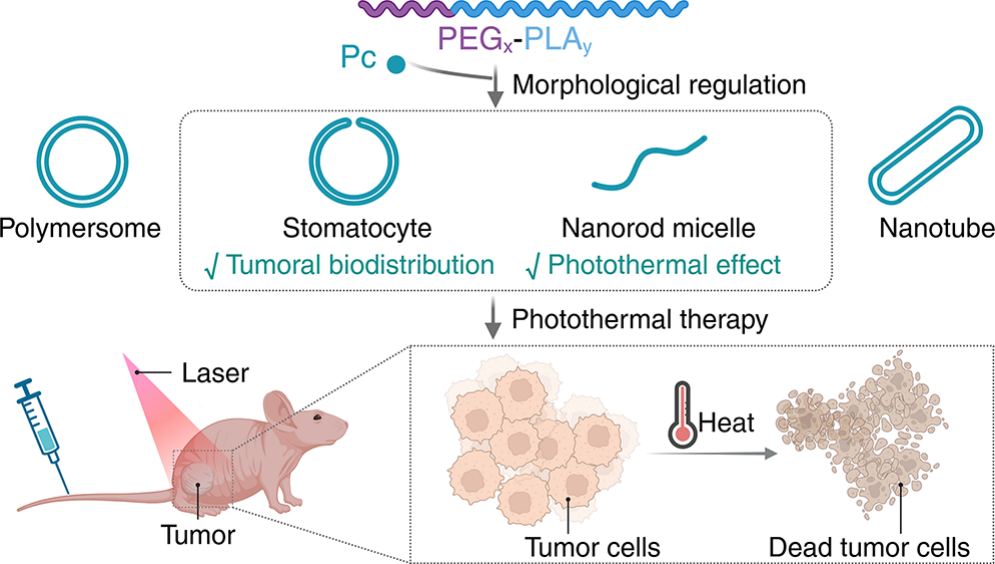

The morphology of nanodrugs is of utmost importance in photothermal therapy because it directly influences their physicochemical behavior and biological responses. However, clarifying the inherent relationship between morphology and the resultant properties remains challenging, mainly due to the limitations in the flexible morphological regulation of nanodrugs. Herein, we created a range of morphologically controlled nanoassemblies based on poly(ethylene glycol)-*block*-poly(d,l-lactide) (PEG–PLA) block copolymer building blocks, in which the model photosensitizer phthalocyanine was incorporated. Four different topologies were compared, namely, spherical vesicles, bowl-shaped vesicles, rodlike micelles, and vesicular tubes. The photothermal properties and *in vivo* tumoral biodistribution were investigated, revealing their relationship with the particle morphology. Finally, the tumor ablation capability of the optimized nanodrugs was demonstrated. This study represents a systematic study of the morphologically discrete regulation of nanodrugs, highlighting the importance of customization of supramolecular photothermal nanodrugs toward clinical antitumor therapy.

## Introduction

Tumor photothermal therapy (PTT) is a cutting-edge, minimally invasive treatment that leverages photothermal agents to selectively target and ablate tumor tissues.^1−5^ Advances in supramolecular chemistry have revolutionized the fabrication of photothermal nanomaterials, such as the emerging small-molecule-based self-assembling nanodrugs.^6−8^ Particularly, it has been demonstrated that the morphology of self-assembled nanodrugs is critically important because it directly influences their physiochemical and biological properties, such as the enhanced photothermal effect of photosensitizers in particulate nanostructures^9^ and prolonged blood circulation of drug molecules imparted by erythrocyte hitchhiking.^10−12^ Regarding this, understanding the relationship between morphology and the resultant properties of photothermal nanodrugs is crucial for their tailored design.

Notwithstanding the considerable availability of morphologically discrete photothermal nanodrugs, clarifying the inherent relationships remains challenging for the following two reasons. First, the existing building blocks used to create nanodrugs have limited morphological diversity due to their fixed chemical structure^13^ and restricted fabrication dynamics.^14^ Additionally, biosafety remains a major hindrance to biological applications owing to the complex and unclear metabolism *in vivo*.^15^ Therefore, it is of importance to employ biocompatible building blocks for assembly that enable flexible control over the discrete morphologies of photothermal nanodrugs and to elucidate the inherent relationships of morphology, photophysical properties, and biological behavior.

**Figure 1:**
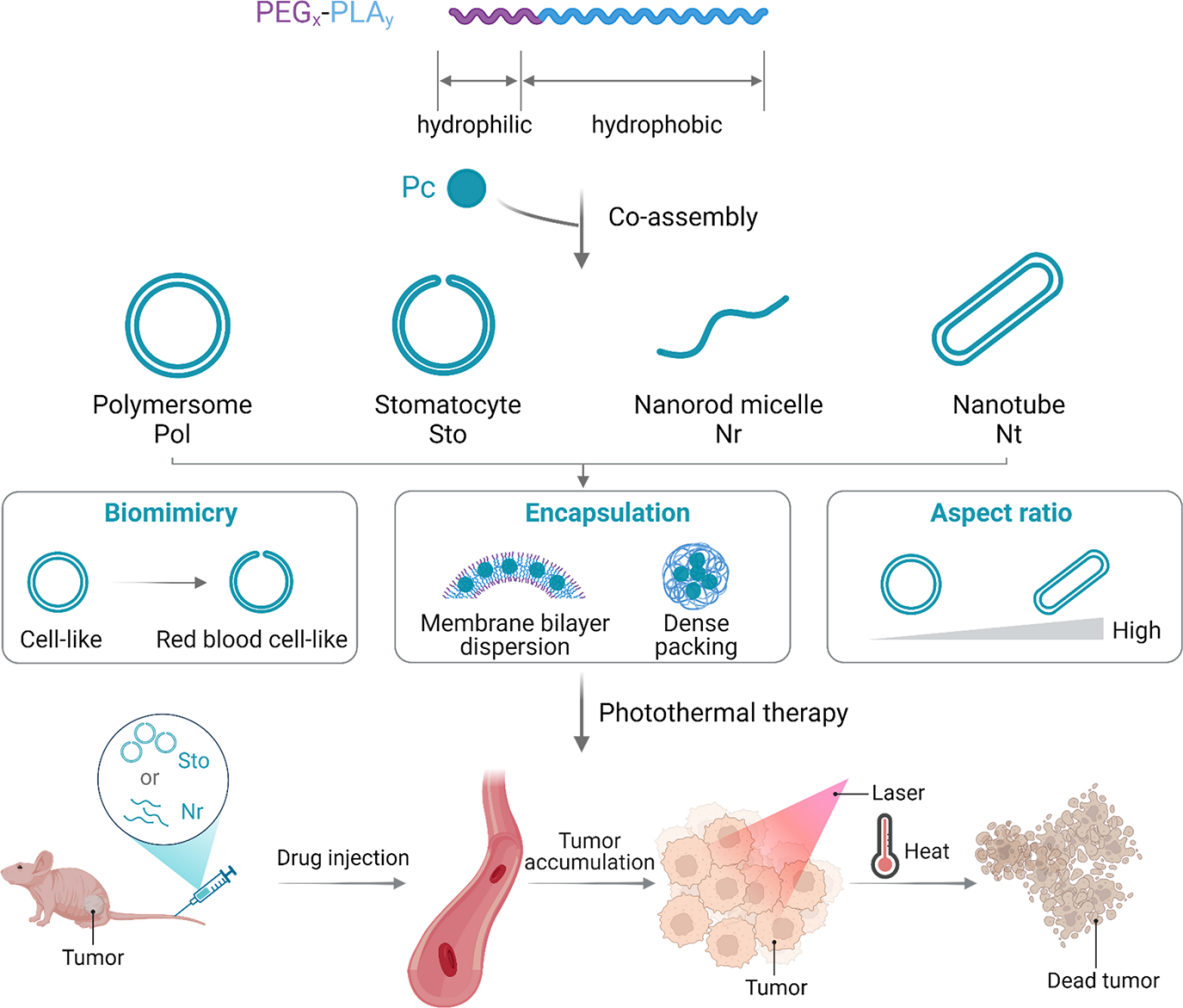
Schematic illustration of morphologically discrete supramolecular nanodrugs and their application in tumor photothermal ablation.

In our group, we have much experience with the biodegradable poly(ethylene glycol)-*block*-poly(d,l-lactide) (PEG–PLA) block copolymer as building block for nanoassemblies. In particular, we have developed a library of nanoparticles based on well-defined PEG–PLA copolymers by controlling polymer composition, such as the length and ratio of hydrophilic/hydrophobic blocks, and processing parameters, such as osmotic pressure equilibration during assembly.^16−18^ In this contribution, we have utilized the supramolecular scaffold formed by PEG–PLA copolymers to spatially organize photothermal phthalocyanines (Pc) as model agents into morphologically discrete nanodrugs. The parameters of these nanodrugs that were varied include spherical vesicles versus red blood cell-shaped ones (polymersomes vs stomatocytes), micellar versus vesicular assembly (nanorods vs tubular vesicles), and aspect ratios (spheres vs nanotubes), which affect their properties such as the loading capacity of Pc and application potential. Through comparative exploration of these nanodrugs, the relationship between the structure, photothermal effect, and *in vivo* tumoral biodistribution was revealed (Figure 1). Considering their optimal photothermal effect and superior tumor accumulation, nanorods and stomatocytes were, respectively, selected, and their capability was demonstrated to ablate tumors effectively. This systematic study therefore provides guidance for the construction and optimization of supramolecular photothermal nanodrugs for clinical antitumor therapy.

## Results and Discussion

### Design and Morphological Characterization of Nanodrugs

The morphology of nanodrugs is of particular interest because parameters such as the particle topology, aspect ratio, and spatial organization of the therapeutic agents directly affect their physiochemical and biological properties. To create a series of nanoparticles in which these parameters could be varied without changing the chemical composition of the scaffold, we employed the biodegradable poly(ethylene glycol)-*block*-poly(d,l-lactide) (PEG–PLA) block copolymer as the building block. Previously, we demonstrated the flexibility of PEG_*x*_-PLA_*y*_ to form different structures by varying the ratio between hydrophilic and hydrophobic domains and the method of assembly.^19^ For this investigation, three kinds of block copolymers were synthesized, namely, PEG_44_-PLA_120_, PEG_44_-PLA_90_, and PEG_22_-PLA_45_ (Figures S1–S3 and Table S1). The amphiphilic block copolymer PEG_44_-PLA_120_ formed polymersomes using the solvent exchange method (Figure S4). Pc was included in the hydrophobic membrane via coassembly (Pol-Pc). Stomatocytes, a type of nanostructure with a concave or bowl-like morphology that highly resembles red blood cells (RBCs) and may benefit from similar advantageous biological properties, such as a long circulation lifespan,^12^ were fabricated through the osmotically induced shape transformation of these PEG_44_-PLA_120_ polymersomes^20^ via dialysis against a 75 mM NaCl solution (Sto-Pc). The aspect ratio plays a crucial role in biological interactions, as higher aspect ratios significantly accelerate particle–cell interactions and cellular internalization of particles.^21^ By shortening the PEG/PLA block to PEG_22_-PLA_45_ and using osmotic pressure-induced shape transformation^22^ upon dialysis against 50 mM NaCl, tubular vesicle nanostructures were formed, which were coassembled with Pc (Nt-Pc). Unlike encapsulation within a vesicle structure, the dense packing of photosensitizers in the hydrophobic core can increase intermolecular collisions of photoactivated photosensitizers, which enhances the photothermal conversion properties.^9^ Accordingly, nanorod morphologies with an elongated, flexible structure were obtained by assembling PEG_44_-PLA_90_; coassembly with Pc yielded the photothermal agent-loaded (Nr-Pc) structures. This morphology also allowed us to investigate the difference in packing between Pc in a bilayer membrane and a micellar core.

To optimize the loading efficiency, we investigated a series of mass ratios between polymers and Pcs upon assembly. Specifically, the concentration of PEG–PLA was fixed at 5 mg mL^–1^, while the concentration of the Pcs was varied from 0, 0.1, and 0.2 mg mL^–1^ to 0.4 mg mL^–1^ (Table S2). As shown in Table S3 and Figure S5, the average hydrodynamic diameter (*D*_h_) of Pol-Pc, Sto-Pc, and Nt-Pc decreased with an increasing Pc concentration. The smallest formulation size was achieved with a polymer concentration of 5 mg mL^–1^ and a Pc concentration of 0.2 mg mL^–1^ due to optimal hydrophobic interactions. However, beyond this optimal mass ratio, the *D*_h_ of the nanoparticles increased, possibly due to the irregular self-aggregation of Pc. For Nr-Pc, the larger *D*_h_ resulted from the encapsulation of Pc within the inner hydrophobic domain of the rodlike micelle, with size distribution becoming uneven at higher Pc concentrations due to self-aggregation. To balance uniform size distribution and optimal photosensitizer content, the mass ratio of polymers/Pcs in Nr-Pc during assembly was optimized to 5 mg mL^–1^/0.2 mg mL^–1^. To evaluate the encapsulation of Pc in nanostructures under the optimized preparation conditions, Pol-Pc was used as an example. Chromatography measurements were performed (Figure S6). After dialysis and three centrifugations, free Pc was effectively removed from Pol-Pc, as confirmed by the absence of a Pc peak in the supernatant after the third wash. Afterward, the Pol-Pc nanodrug was dissolved in THF and the presence of Pc was observed, demonstrating the successful coassembly of polymers and Pcs.

**Figure 2:**
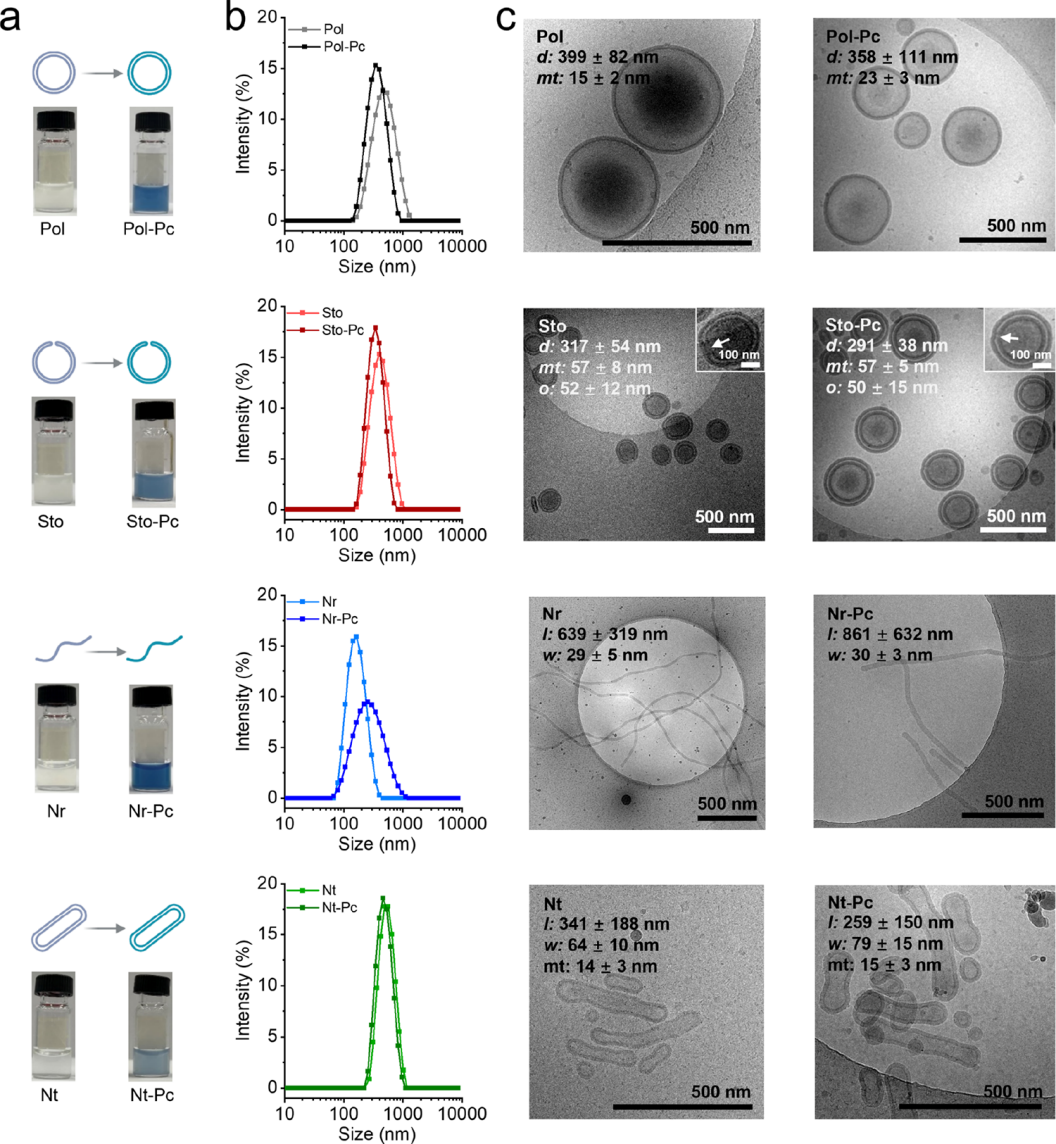
Size and morphological characterization of nanodrugs. (a) Optical pictures demonstrating the encapsulation of phthalocyanine (Pc), (b) DLS results, and (c) cryo-TEM images of polymer self-assemblies and polymer/Pc coassembled nanodrugs. In figure (c), the diameter is abbreviated as *d*, membrane thickness is abbreviated as *mt*, length is abbreviated as *l*, width is abbreviated as *w*, and opening is abbreviated as *o*. The white arrows indicate the opening of the Sto-based structures.

The morphology of the four types of nanoparticles, both with and without Pc, was characterized by dynamic light scattering (DLS) and cryogenic transmission electron microscopy (cryo-TEM) (Figure 2). From the DLS results, it could be concluded that particles were formed with narrow polydispersity. Furthermore, the effect of Pc loading was only minor except for the nanorod system (Figure 2b). This was confirmed by cryo-TEM images (Figure 2c). PEG_44_-PLA_120_-based polymersomes had a diameter of 399 ± 82 nm and a membrane thickness of 15 ± 2 nm. After coassembly with Pc, their diameter and membrane thickness changed to 358 ± 111 and 23 ± 3 nm, respectively. For PEG_44_-PLA_120_-based stomatocytes, the diameter changed from 317 ± 54 to 291 ± 38 nm, whereas the opening gap showed little change from 52 ± 12 to 50 ± 15 nm, and the membrane thickness remained 57 nm after coassembly with Pc. The nanorods changed in length from 693 ± 319 to 861 ± 632 nm and the width changed from 29 ± 5 to 30 ± 6 nm when Pc was coassembled. Finally, the nanotubes changed in length from 341 ± 188 to 259 ± 150 nm and in width from 64 ± 10 to 79 ± 15 nm, whereas the membrane thickness remained similar (from 14 ± 3 to 15 ± 3 nm) upon Pc coassembly. Based on the DLS and cryo-TEM results, a general trend observed with the vesicle-based nanodrugs, Pol-Pc, Sto-Pc, and Nt-Pc, was a decreased size compared to their unencapsulated nanostructures. This could be due to the hydrophobic arrangement of Pc within the membrane bilayer structure, resulting in more compact structures. In contrast, Nr-Pc showed an increase in size compared to that of its unencapsulated counterpart, likely due to its flexibility and ability to accommodate more cargo.

The encapsulation efficiency (EE), a key parameter describing the drug properties,^23^ was measured. As summarized in Table S5, the highest Pc EE was found in Nr-Pc, with a calculated value of 39%, which is in line with the observed increase in the size of the nanorods. The EEs of the other three vesicle-based nanodrugs were calculated as 26% for Pol-Pc, 19% for Sto-Pc, and 11% for Nt-Pc, respectively. The lower encapsulation might be caused by the bilayer membrane structure, which provides limited space to incorporate hydrophobic drug molecules. Regarding the other three vesicle-based nanodrugs, the EEs of Pol-Pc and Sto-Pc should ideally be similar since the latter is shape-transformed from the former. However, the lower EE of Sto-Pc could be attributed to a greater loss of Pc induced by a higher osmotic pressure during dialysis. Additionally, despite the high aspect ratio and increased surface area, which could enhance drug encapsulation,^24^ Nt-Pc still showed an inferior value compared to Pol-Pc and Sto-Pc. This might be due to the shorter hydrophobic PLA chain of the copolymer building blocks.

### Photophysical Properties of Nanodrugs

**Figure 3:**
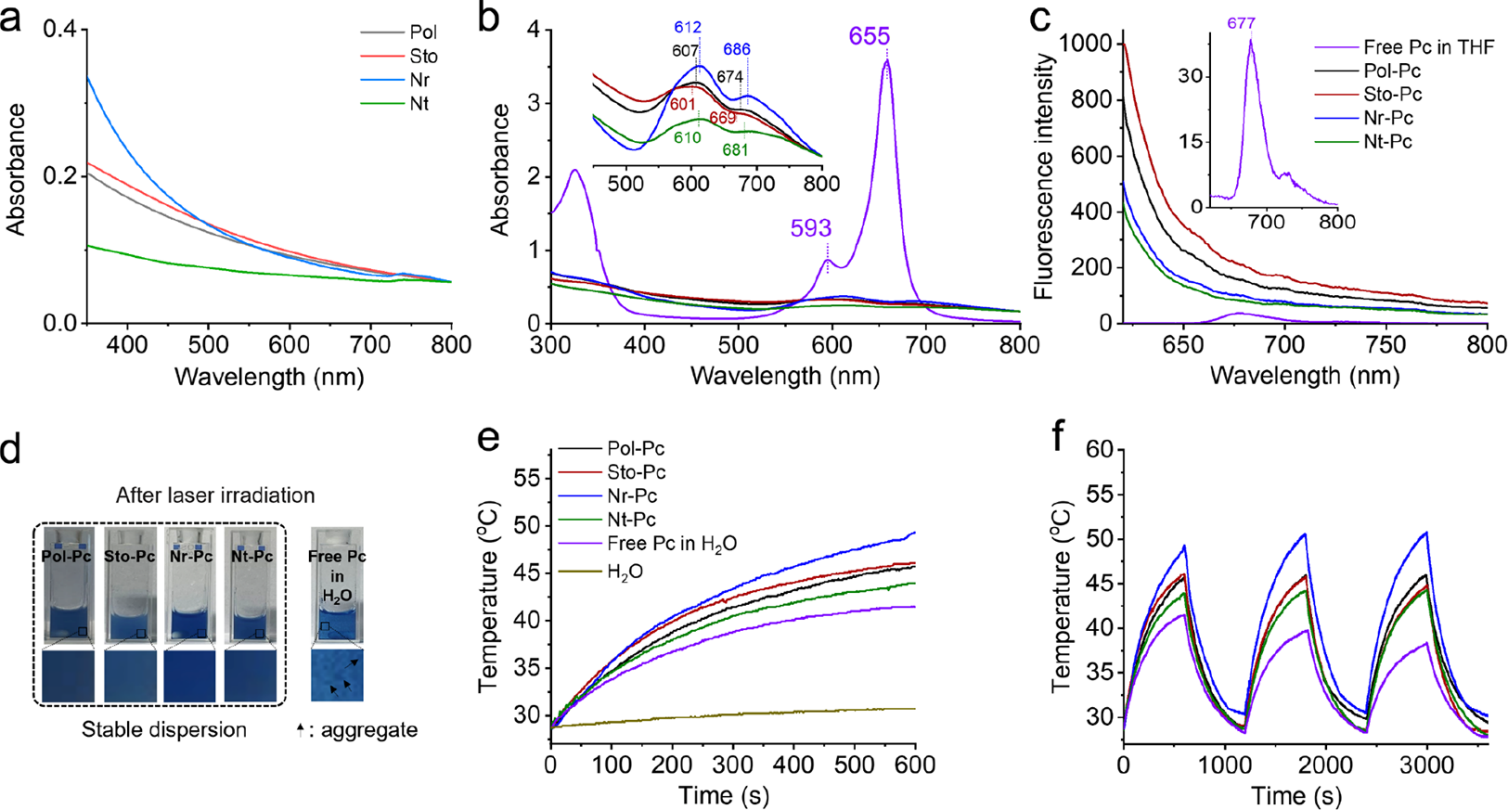
Photophysical properties of nanodrugs. (a) Absorption spectra of nanoparticles without Pc. (b) Absorption spectra and (c) fluorescence spectra of nanodrugs. The zoomed-in inset in part (b) indicates the details of the Q bands of Pc within the four types of nanodrugs, and the zoomed-in inset in part (c) indicates the detailed fluorescence of Pc. The concentration of Pc used in figures (a–c) is 0.01 mg mL^–1^. (d) Optical picture of nanodrugs after laser irradiation. (e) Temperature elevation profiles and (f) continuous irradiation–cooling profiles of the nanodrugs. The concentration of Pc used in figures (d–f) is 0.02 mg mL^–1^.

Since the organization of photosensitive molecules within nanostructures significantly influences their photophysical and chemical features, these properties of Pc in the different nanodrugs were systematically investigated. The absorption spectra of the nanostructures without Pc showed no characteristic peak in the wavelength range of 500–750 nm (Figure 3a), indicating no spectral interference from the polymer assemblies. As shown in Figure 3b and Table S4, the characteristic Q band of Pc^25^ located at 593 and 655 nm exhibited a bathochromic shift when coassembled with polymers. The degree of the shift followed the sequence Nr-Pc > Nt-Pc > Pol-Pc > Sto-Pc. According to the Kasha’s rule,^26^ this suggests that Pcs were stacked in a J-aggregation mode within these four types of nanodrugs. Obviously, the larger shift observed in Nr-Pc compared to the other three nanodrugs was attributed to the denser aggregation of Pcs in the hydrophobic core of the nanorods, causing more orbital overlap.^27,28^ The aggregation, mainly due to the shortened intermolecular distance, might be influential to the radiative decay pathway once the photosensitive molecules are photoactivated. Therefore, comparative fluorescence spectra were also studied (Figure 3c). Compared to the fluorescence emission of Pc at the maximum peak at λ_max_ = 677 nm, the four types of nanodrugs showed a fluorescence quenching effect. To demonstrate that this effect was indeed caused by the coassembly with polymers, a control experiment was conducted in which Pc was released from the assembly using a freeze-drying and redissolution procedure. In the absorption spectra (Figure S7a) of Pc extracted from all four types of nanodrugs, indeed, the characteristic absorption peak of monomeric Pc was observed. Additionally, Figure S7b shows the restored fluorescence emission of Pc, similar to the monomeric Pc molecules without coassembly. The collective results indicated that the bathochromic shift in absorption spectra and the fluorescence quenching were both caused by the coassembly with polymers.

The coassembly with polymers promoted the aggregation of Pcs and shortened their intermolecular distance, enhancing their interaction in the excited state, which made the system conducive to photothermal conversion.^29^ Therefore, the obtained nanodrugs were expected to be photothermally active, and their photothermal conversion properties were next investigated. The nanodrugs were irradiated with a 660 nm laser. After irradiation, the nanodrugs maintained their original dispersed state (Figure 3d), indicating that the nanostructures improved the stability of Pcs. In contrast, Pcs dispersed in water formed large aggregates upon irradiation. Both this aqueous dispersion and the four types of nanodrugs effectively raised the temperature through laser irradiation, with a calculated Δ*T* of over 15 °C. Importantly, the temperature-raising effect of the nanodrugs was more significant than that of Pcs dispersed in water, likely because of the increased stability (Figure 3e). Furthermore, a heating–cooling cycle was performed to indicate the photothermal conversion stability (Figure 3f). Within three cycles, the maximum temperature reached by Pcs dispersed in water gradually decreased due to the limited stability of the dispersion. However, no significant differences were observed within three cycles for the four types of nanodrugs, proving their outstanding photothermal conversion stability. Moreover, the photothermal conversion efficiency (η) was calculated and ranked as Nr-Pc 45.7% > Nt-Pc 43.3% > Pol-Pc 42.2% > Sto-Pc 41.6% (Table S6), which correlated with the degree of the bathochromic shift. The results indicate that the photothermal conversion and ability of Pcs can be improved by coassembly with polymers: the more pronounced the bathochromic shift, i.e., the smaller the intermolecular distance among Pcs, the higher the photothermal conversion efficiency achieved.

### *In Vitro* Photothermal Evaluation of Nanodrugs

**Figure 4:**
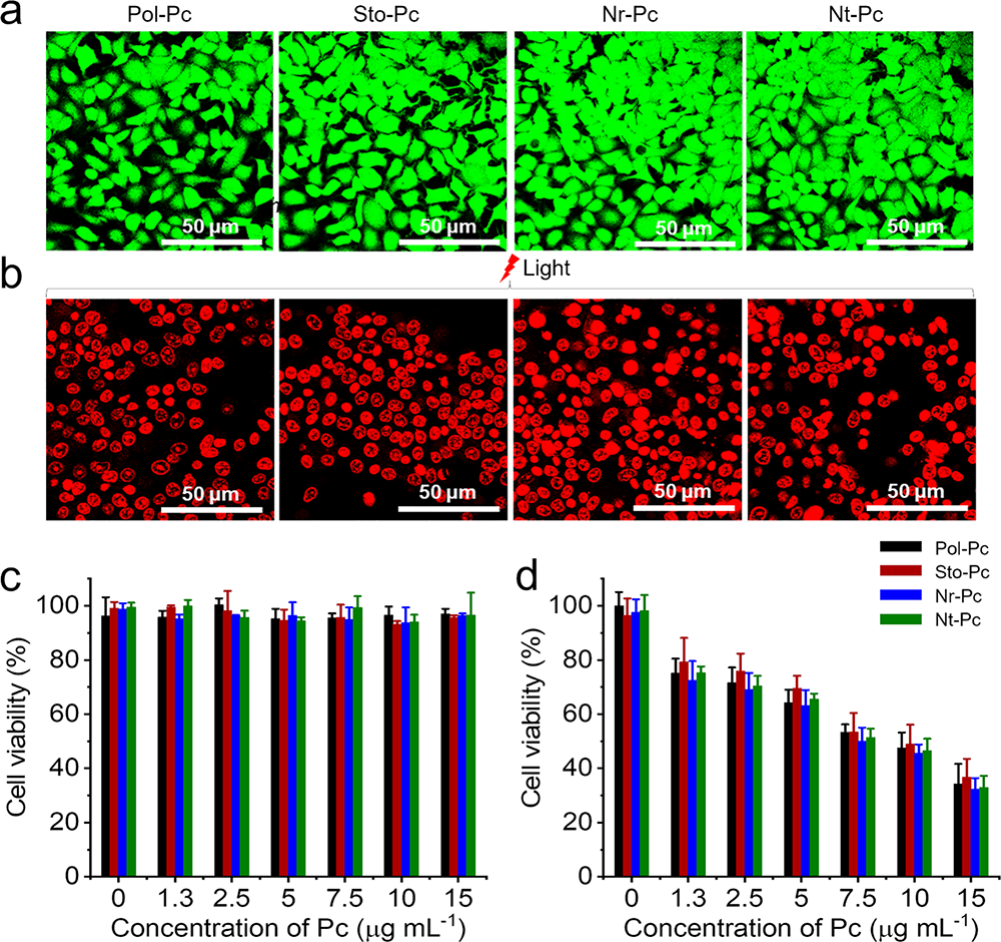
*In vitro* photothermal evaluation of nanodrugs. Confocal laser scanning microscopy (CLSM) images of MCF-7 cells stained with calcein-AM/PI dyes before (a) and after (b) 660 nm laser irradiation (1 W cm^–2^, 20 min). Before staining, the MCF-7 cells were incubated with the four nanodrugs at a Pc concentration of 0.015 mg mL^–1^ for 24 h. MTT assay of MCF-7 cell viability after exposure to different concentrations of nanodrugs (c) without or (d) with 660 nm laser irradiation (1 W cm^–2^, 5 min). The error bars represent the standard deviation (*n* = 6).

For subsequent biological applications, the stability of the nanodrugs was first tested in a PBS buffer to mimic physiological conditions, and their size changes were monitored by DLS over time (Figure S8). No significant changes were observed within 2 weeks, demonstrating that these nanodrugs possessed excellent stability in physiological media, laying the foundation for further biological applications. Thereafter, the photothermal ablation effect of the nanodrugs was evaluated *in vitro*. The human breast cancer MCF-7 cells were cultured and, respectively, incubated with the four types of nanodrugs for 24 h. The calcein AM/PI live/dead staining method was used to characterize the cells.^30^ As shown in Figure 4a, cells in all four groups maintained strong green fluorescence without laser irradiation, indicating no damage to the cells. However, after illumination, red fluorescence appeared within the cells in all four groups (Figure 4b), demonstrating that photothermal ablation induced cell necrosis or apoptosis under laser irradiation. Then, the photothermal ablation effect of the four types of nanodrugs was quantitatively measured using the standard (3-(4,5-dimethylthiazol-2-yl)-2,5-diphenyltetrazolium bromide) (MTT) assay. Without laser irradiation, cell activity was unaffected despite the coincubation with the nanodrugs, as shown in Figure 4c, indicating the excellent biocompatibility of the nanodrugs. However, the cell viability decreased after laser irradiation. Moreover, the higher the concentration of the nanodrugs added, the lower the cell survival rate. In particular, cell activity was reduced below 50% when the nanodrug concentration reached 10 μg mL^–1^ (Figure 4d). These results indicate that the nanodrugs possess an excellent photothermal ablation capacity, which is proportional to the added drug concentration, making them exploitable for antitumor therapy.

### *In Vivo* Tumoral Biodistribution of Nanodrugs

**Figure 5:**
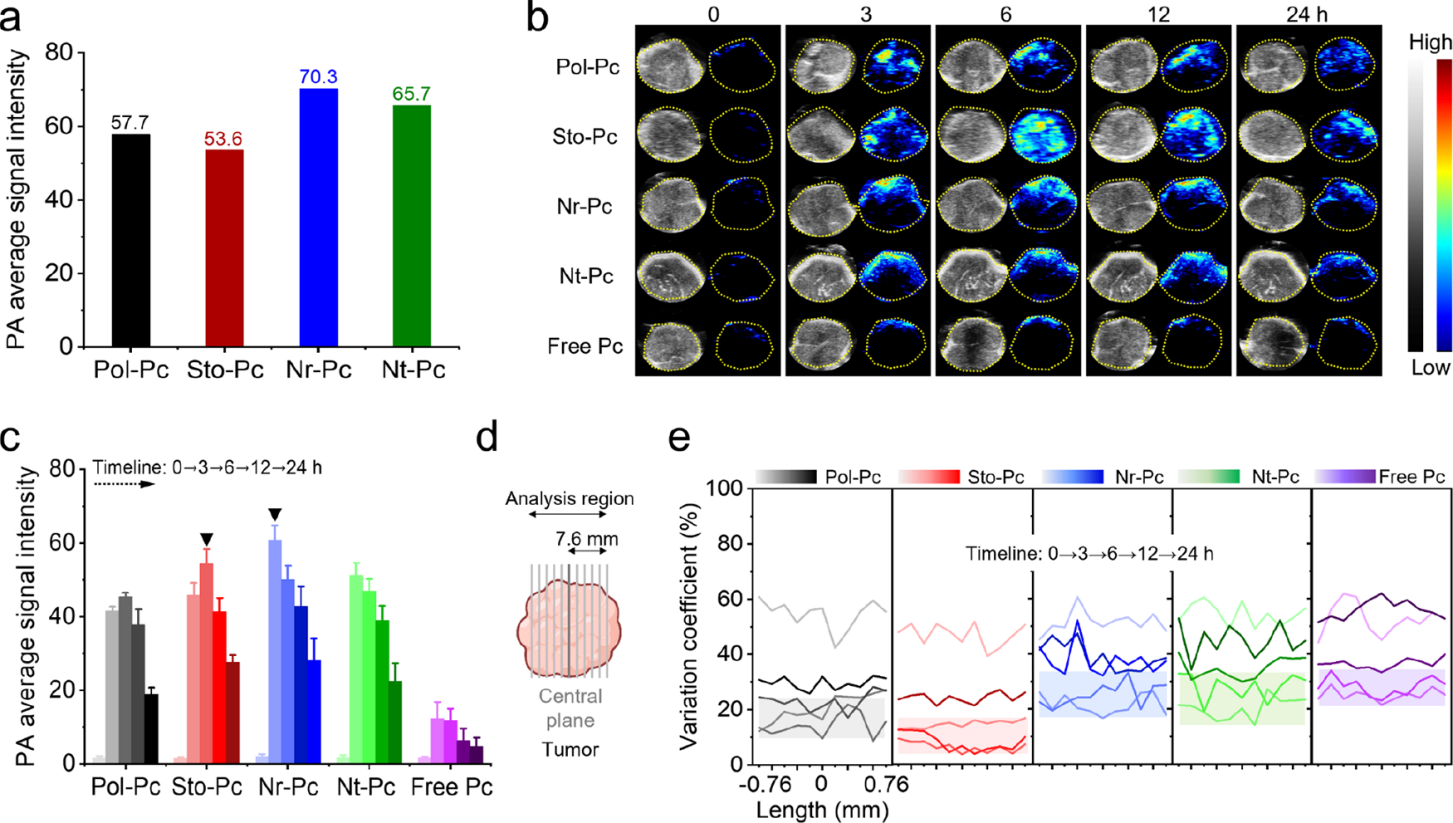
Tumoral biodistribution of nanodrugs. (a) Photoacoustic (PA) average signal of the four types of nanodrugs. (b) PA images of mice bearing MCF-7 tumors over time. The mice were intravenously injected with the four types of nanodrugs with a Pc dosage of 0.05 mg mL^–1^, 200 μL. The left panel indicates the ultrasound images, and the right panel indicates the PA images. The area marked by a yellow line indicates the tumor tissue. (c) Quantitative analysis of PA average signal at different time intervals. The darkness of the lines corresponds to the timeline from 0 to 24 h (from light to dark). The error bars represent the standard deviation (*n* = 6). (d) Schematic illustration of CV value analysis within tumor tissues. (e) CV value analysis of PA signals within tumor tissues in different groups. The darkness of the lines corresponds to the timeline from 0 to 24 h, while the colored region highlights the value range within the time interval between 3 and 6 h.

Taking advantage of the photothermal effect, the tumoral biodistribution of the nanodrugs was easily monitored by photoacoustic (PA) imaging technology.^31^ The PA signals of the nanodrugs loaded with the same Pc concentration were ranked as follows: Nr-Pc 70.3 > Nt-Pc 65.7 > Pol-Ps 57.7 > Sto-Pc 53.6 (Figure 5a), which aligns with their photothermal conversion efficiencies. To evaluate the *in vivo* tumoral biodistribution of the nanodrugs, a mouse subcutaneous tumor model inoculated by MCF-7 cells was established. After intravenous injection of the respective nanodrugs, the PA signal within the tumor sites was monitored at different time intervals (Figure 5b,c). Before injection (0 h), no significant PA signal was detected at the tumor sites in any of the groups. It is worth noting that free Pcs without encapsulation exhibit low tumor targeting and retention due to rapid excretion from the body via the reticuloendothelial system.^32^ The nanodrugs exhibited an enhanced permeability and retention effect throughout the observation period.^33^ Specifically, at 3 h postinjection, the PA signal intensity in the tumor tissue of the Nr-Pc group was higher than that in the other three groups. This could be attributed to its higher photothermal conversion efficiency and smaller size, which facilitated more efficient endocytosis.^34^ Similarly, the PA signal intensity in the Nt-Pc group peaked at 3 h postinjection. The faster transport to the tumor site observed in these two groups compared to the Pol-Pc and Sto-Pc groups may be due to their long aspect ratios, which allowed them to align parallel to the direction of blood flow, resulting in faster circulation.^11^ For the Pol-Pc and Sto-Pc groups, the maximum PA signal intensity was observed at 6 h postinjection, with an extended retention time. This may be due to their biomimetic structures, which likely helped them evade clearance by the reticuloendothelial system *in vivo*.^35^ Notably, the Sto-Pc group exhibited the highest PA signal intensity at 6 h postinjection and, more interestingly, demonstrated superior tumor penetration. To quantify the drug distribution within the tumor, the coefficient of variation (CV) value was used to describe the variability of the PA signals relative to the mean in tumor cross sections (Figure 5d,e). Within the 3–6 h time interval postinjection, the maximum of the CV value in the Sto-Pc group was below 17%, which was significantly lower than the other three groups of Pol-Pc with 22%, Nr-Pc with 34%, and Nt-Pc with 33%, indicating a more uniform drug distribution within the tumor tissue. This improved distribution could be attributed to the nanostructure of Sto-Pc, which mimics the flexibility of red blood cells. This flexibility allows Sto-Pc to navigate narrow capillaries and complex vascular networks, thereby promoting prolonged blood circulation and deeper tumor penetration.

### *In Vivo* Photothermal Evaluation of Nanodrugs

**Figure 6:**
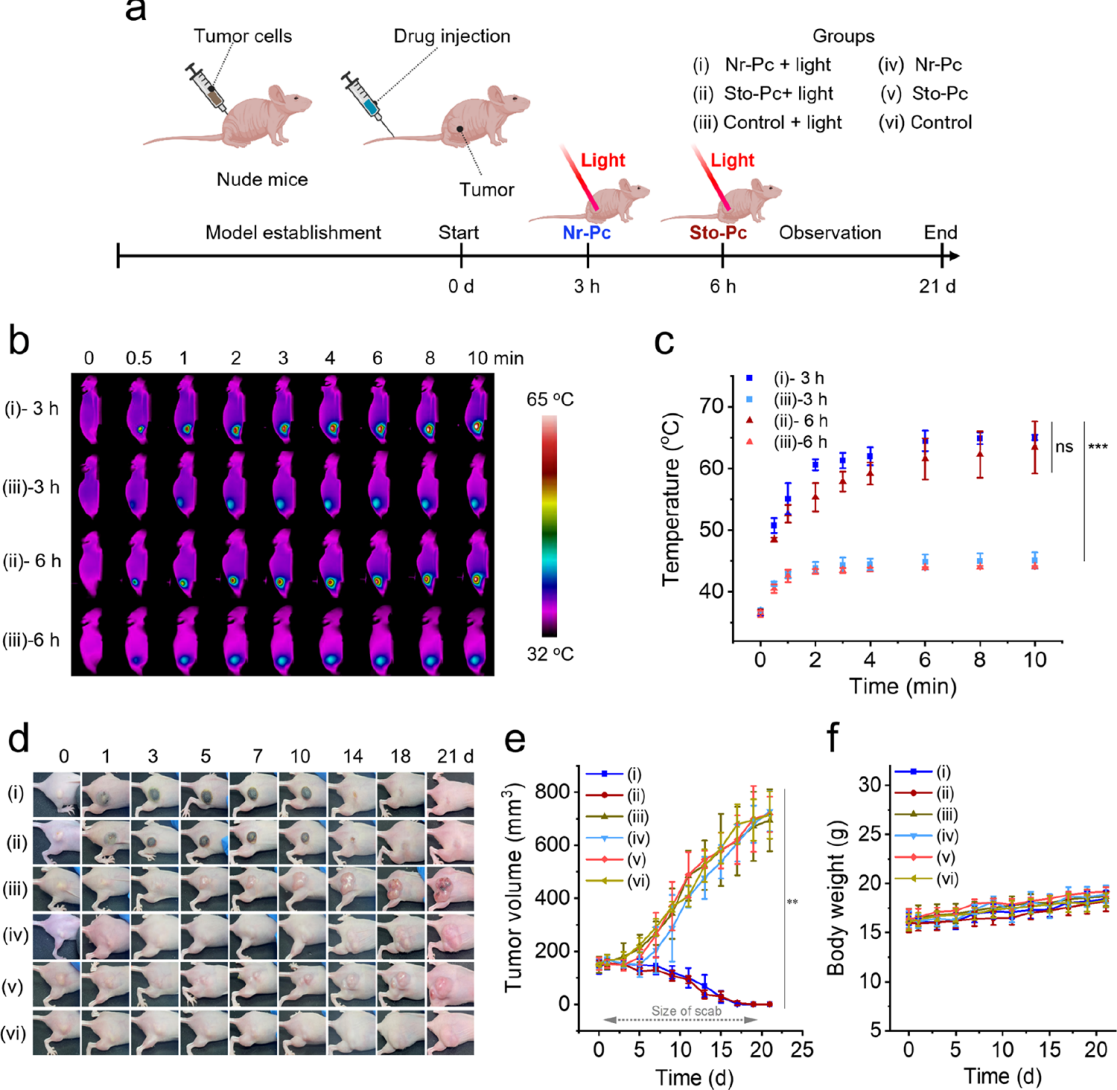
PTT evaluation of the nanodrugs. The groups were as follows: (i) Nr-Pc + laser, (ii) Sto-Pc + laser, (iii) Control + laser, (iv) Nr-Pc, (v) Sto-Pc, and (vi) Control. Each group consisted of 6 mice, except for group (iii), which included 12 mice. In this group, 6 mice served as controls for (i) with laser irradiation at 3 h postinjection, while the other 6 mice served as controls for (ii) with laser irradiation at 6 h postinjection. (a) Schematic diagram of mouse model establishment and PTT therapy. (b) Representative thermal images of mice bearing MCF-7 tumors treated with (i) Nr-Pc (Pc dosage: 0.05 mg mL^–1^, 200 μL), (ii) Sto-Pc (Pc dosage: 0.05 mg mL^–1^, 200 μL), and their corresponding (iii) control groups (5% glucose, 200 μL) under laser irradiation (660 nm, 1 W cm^–2^, 10 min) at the preset time points. (c) Temperature profiles of tumor sites during laser irradiation. Error bars represent standard deviation (*n* = 6). (d) Representative images of mice bearing MCF-7 tumors at different time intervals after therapy. (e) Tumor volume curves and (f) body weight profile of mice after treatment. Error bars represent standard deviation (*n* = 6). ns: not significant (*p* > 0.05), *p < 0.05, **p < 0.01, and ***p < 0.001.

In view of the high photothermal effect of Nr-Pc and the superior tumoral biodistribution of Sto-Pc, they were selected as the model groups to evaluate photothermal ablation *in vivo*. After establishing tumor-bearing mouse models, they were divided into six groups: (i) Nr-Pc + laser group, (ii) Sto-Pc + laser group, (iii) Control + laser group, (iv) Nr-Pc group, (v) Sto-Pc group, and (vi) Control group. Nanodrugs including Nr-Pc and Sto-Pc and PBS (control group) were respectively injected into the mice through the tail vein. At the therapeutic window determined by PA imaging *in vivo*, the mouse tumors were irradiated accordingly. In the Nr-Pc group, irradiation was conducted at 3 h postinjection, while in the Sto-Pc group, it was 6 h postinjection. Changes in tumor growth and body weight of the mice were monitored over time (Figure 6a). As shown in Figure 6b,c, the average tumor temperature of the mice in the Nr-Pc and Sto-Pc groups increased to 65.0 and 63.4 °C within 10 min, respectively, while the Δ*T* of mouse tumors in the control group only increased by ∼7 °C. These results indicate that the nanodrugs concentrated at the tumor site led to a rapid temperature increase under laser irradiation. Subsequently, tumor tissues in the (i) Nr-Pc + laser group and (ii) Sto-Pc + laser group showed a regular tumor ablation process including swelling, blackening, scabbing, falling off, and returning to normal skin status, with no tumor recurrence observed during the monitoring period. In contrast, tumors in the (iii) Control group and the other three groups (iv–vi) without laser irradiation gradually grew. Additionally, no discernible changes were observed in the four groups (iii–vi) at the end of the observation period (Figure 6d,e). The results suggest that only mice injected with nanodrugs and treated with laser irradiation had their tumors ablated effectively. The body weight of the mice in all groups showed a gradual increase, proving the biosafety of the nanodrugs and the treatment modality. Moreover, hematoxylin and eosin (H&E) staining images showed no tumor metastasis in the major organs of the mice in the (i) Nr-Pc + laser group and (ii) Sto-Pc + laser group, whereas other tumor lesions were observed in the other four groups (iii–vi) (Figure S9). This verifies that nanodrugs photothermally ablated tumors without causing damage to other organs.

## Conclusions

In summary, we designed four morphologically discrete nanodrugs by coassembling PEG–PLA block copolymers with phthalocyanines (Pc) as the model photothermal agent. The particle shape, aspect ratio, and spatial positioning of Pc in these nanodrugs were compared, regarding their photothermal properties and *in vivo* tumoral distribution. Nr-Pc, with the highest photothermal conversion efficiency, and Sto-Pc, with the best tumor distribution, were further selected and demonstrated their biocompatibility and efficiency in antitumor therapy.

By flexible molecular design and controllable self-assembly, photothermal nanodrugs with diverse morphologies can be easily constructed. The relationship between supramolecular structure and application properties was elucidated, providing guidelines for drug customization. For example, the structure with densely hydrophobic packing of photothermal agents benefits the photothermal effect not only because of the high drug encapsulation but also due to increased intermolecular collision. Nanodrugs with a biomimicry of red blood cells show excellent biodistribution, as they prolong blood circulation and uniform tumoral dispersion. Overall, this study holds promise for constructing supramolecular photothermal nanodrugs with specific needs.

## Methods

### Nanodrug Preparation

The mixed solvent of dioxane/THF (v/v = 4/1) was used to dissolve the polymer to obtain a solution (50 mg mL^–1^), and THF was used to dissolve Pc to obtain a solution (0.5 mg mL^–1^) for the following nanodrug preparation.

### Polymersomes-Pc (Pol-Pc) Preparation

A 100 μL portion of PEG_44_-*b*-PLA_120_ solution (50 mg mL^–1^) was mixed with 400 μL of Pc solution (0.5 mg mL^–1^). Subsequently, 1.5 mL of pure water was added by a syringe pump at a dropwise rate of 1 mL h^–1^. Next, the resulting suspension was transferred to a dialysis bag, which was placed in pure water for dialysis at 4 °C for 24 h with a water change after 1 h. All of the above procedures were performed with stirring. Finally, the Pol-Pc suspension was centrifuged at a relative centrifugal force of 6010*g* (8000 rpm) for 20 min. The upper supernatant was discarded, and the precipitated Pol-Pc nanodrug retained in the tube was resuspended in pure water. This centrifugation and washing procedure was repeated three times.

### Stomatocytes-Pc (Sto-Pc) Preparation

The preparation of Sto-Pc was the same as that described for Pol-Pc, with modified dialysis conditions. The dialysis bag with the obtained suspension was placed in 75 mM NaCl solution for dialysis at 4 °C for 24 h, with a water change after 1 h. All the above procedures were performed with stirring. Finally, the centrifugation and washing procedure of Sto-Pc was the same as for Pol-Pc.

### Nanorods-Pc (Nr-Pc) Preparation

One hundred μL of PEG_44_-*b*-PLA_90_ solution (50 mg mL^–1^) was mixed with 400 μL of Pc solution (0.5 mg mL^–1^). Subsequently, 1.5 mL of pure water was added by a syringe pump at a dropwise rate of 1 mL h^–1^. Next, the resulting suspension was transferred into a dialysis bag, which was placed in pure water for dialysis at 4 °C for 24 h with a water change after 1 h. All the above procedures were performed with stirring. Finally, the centrifugation and washing procedure of Nr-Pc was the same as that for Pol-Pc.

### Nanotubes-Pc (Nt-Pc) Preparation

One hundred μL of PEG_22_-*b*-PLA_45_ solution (50 mg mL^–1^) was mixed with 400 μL of Pc solution (0.5 mg mL^–1^). Subsequently, 1.5 mL of pure water was added by a syringe pump at a dropwise rate of 1 mL h^–1^. Next, the resulting suspension was transferred into a dialysis bag, which was placed in 50 mM NaCl solution for dialysis over 24 h with a water change after 1 h. All the above procedures were performed with stirring. Finally, the centrifugation and washing procedure of Nt-Pc was the same as for Pol-Pc.

### Polymer-Based Self-Assemblies without Pc

A 100 μL polymer solution (50 mg mL^–1^) was mixed with 400 μL of THF solution. Subsequently, 1.5 mL of pure water was added by a syringe pump at a dropwise rate of 1 mL h^–1^. Next, the resulting suspension was transferred into a dialysis bag with a molecular weight cutoff of 12–14 kDa, which was placed under the same conditions as the corresponding photosensitive nanodrug preparations for dialysis over 24 h. All the above procedures were performed with stirring, and the centrifugation and washing procedure for these polymer-based self-assemblies was the same as that used for their corresponding coassemblies with Pc.

### Chromatography Measurements of Pol-Pc

During the three centrifugations, the upper supernatant was collected for measurement to determine the purification efficiency of unencapsulated Pc. The final precipitated Pol-Pc nanodrug retained in the tube was freeze-dried and dissolved in THF. The control groups without Pc, including the THF solvent and Pc and Pol self-assemblies, were studied as well. All measurements were performed using GPC chromatograms obtained from the UV detector.

### Determination of Encapsulation Efficiency (EE) of Pc in Nanodrugs

During the third centrifugation of the nanodrugs, the upper supernatant was discarded and the precipitates (nanodrugs) retained in the tube were redissolved in THF. These were then analyzed using UV–vis spectroscopy. The Pc concentration in the sediment was determined by using the standard curve correction method. The Pc EE was calculated according to the following formula:
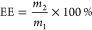
where “*m*_2_” is the weight of the Pc in nanodrugs and “*m*_1_” is the weight of the Pc added during the coassembly.

### Photothermal Performance of Nanodrugs

One mL of a nanodrug suspension (Pc concentration of 0.02 mg mL^–1^) was placed in a quartz cuvette and then irradiated by a 660 nm laser with a power density of 1 W cm^–2^ for 10 min at room temperature (25 °C). A 1 mL portion of free Pc dispersed in pure water (0.25 mg mL^–1^) and 1 mL of pure water were used as control groups. The temperature was recorded every 1 s with a digital thermometer.

Continuous irradiation–cooling profiles for indicating the photothermal conversion stability were tested in a temperature increase and decrease mode. The irradiation time and the cooling time were 10 min, respectively, and the total circulation number was 3.

The photothermal conversion efficiency (η) of the NPs was calculated by following a standard formula:
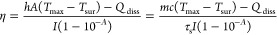
where the “*T*_max_” and “*T*_sur_” are the initial and the highest temperature of nanodrug dispersions, respectively. “*Q*_diss_” is measured by a Spectra-Physics power meter representing the heat dissipation. “*I*” denotes the power of the laser. “*A*” is the absorbance at 660 nm. “*m*” denotes the quality of the tested solution. “*c*” is the specific heat capacity of the solvent (pure water). The value of “τ_s_” was calculated using the following formula.
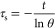
“θ” is the dimensionless driving force, and “*t*” is the corresponding time.

### Stability Test of Nanodrugs

The nanodrugs were placed in physiological PBS, and then, they were analyzed with DLS over time. The recording times were 1, 3, 5, 7, and 10 days.

### *In Vitro* Photothermal Ablation of Nanodrugs

Human breast cancer (MCF-7) cells were cultured in a DMEM medium containing 10% FBS (v/v) and 1% penicillin–streptomycin (v/v), where the ambient temperature was 37 °C, the CO_2_ content was 5%, and the humidity was 95%.

To directly test the photothermal ablation of nanodrugs *in vitro*, MCF-7 cells were placed in 35 mm glass-bottom Petri dishes (6 × 10^4^ cells cm^–2^) and cultured for 24 h. Afterward, four kinds of nanodrugs (Pc concentration of 0.015 mg mL^–1^) were added to the dishes for coincubation for 24 h. Then, the cells were washed three times with PBS and fixed with 4% paraformaldehyde. The cells were irradiated with a 660 nm laser with a power density of 1 W cm^–2^ for 20 min. After that, the calcein AM and PI dyes were simultaneously added to the dishes and allowed to dry the cells for 15 min. Imaging was conducted by CLSM, living cells were stained green by calcein AM and excited at 488 nm, and dead cells were stained red by PI and excited at 559 nm.

The standard (3-(4,5-dimethylthiazol-2-yl)-2,5-diphenyltetrazolium bromide) (MTT) assay was used to quantify the effect of photothermal therapy *in vitro*. MCF-7 cells were seeded in 96-well plates (1 × 10^4^ cells well^–1^) and incubated for 24 h. Four kinds of nanodrugs with different concentrations (Pc concentration of 0–15 μg mL^–1^) were added to the 96-well plate for coincubation for 24 h. After that, cells were irradiated through a 660 nm laser with a power density of 1 W cm^–2^ for 5 min, followed by culture for 24 h. Finally, the cell viability was determined using the MTT method. Except for the illumination step, the cells were treated with the same procedures as described above to test the dark toxicity of the four kinds of nanodrugs.

### Photoacoustic (PA) Imaging of Nanodrugs

Four kinds of nanodrugs (Pc concentration of 0.05 mg mL^–1^) were placed in the test tubes, and the signals were obtained with PA imaging equipment, wherein the excitation wavelength was fixed at 680 nm.

### Establishment of the Subcutaneous Xenograft Tumor Model

All animal experiments were conducted in accordance with the protocols approved by the local ethical committee in compliance with the Chinese law on experimental animals. Female BALB/c-nude mice were kept in an environmentally controlled animal facility with a 12/12 cycle. The human xenograft model was created by inoculating 100 μL of the MCF-7 cell suspension (5.0 × 10^7^ cells mL^–1^) in the subdermal dorsal area of mice. Approximately 1 week after inoculation, the tumors were well-established.

### *In Vivo* PA Imaging

After the tumor model was established, 200 μL volumes of 5% glucose suspensions of four kinds of nanodrugs (Pc concentration of 0.05 mg mL^–1^) were injected into the mice through the tail vein. Additionally, a 5% glucose solution of free Pc was used as the control group. Then, the tumor tissues of mice were scanned by PA imaging equipment at different time intervals (0, 3, 6, 12, and 24 h), wherein the excitation wavelength was fixed at 680 nm. The acquired images were analyzed with the equipped software. The coefficient of variation (CV) value was calculated by the following formula.
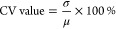
“σ” is the standard deviation of the data, and “μ” is the mean of the data.

### *In Vivo* Photothermal Ablation

After the tumor model was established, 200 μL of Nr-Pc (Pc concentration of 0.05 mg mL^–1^), Sto-Pc (Pc concentration of 0.05 mg mL^–1^), and 5% glucose (control group) was injected into the mice through the tail vein. For the Nr-Pc + laser group, the mouse tumors were irradiated by a 660 nm laser with a power density of 1 W cm^–2^ for 10 min at the 3 h post injection point, while for the Sto-Pc + laser group, the irradiation was conducted at 6 h postinjection. The mouse tumors in the Control + laser group were irradiated correspondingly at 3 or 6 h postinjection. During this process, an infrared thermal imaging camera was used to monitor the temperature change of the mouse tumors. For the groups Nr-Pc, Sto-Pc, and Control group, no laser irradiation was performed.

After the treatment, the body weight and tumor volume of the mice were monitored in the observation period. The tumor volume was calculated using the following formula:
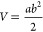
“*a*” indicates the length of the tumor, and “*b*” indicates the width of the tumor.

### Hematoxylin and Eosin (H&E) Staining

After the treatment and observation period, the mice were sacrificed by dislocation, and mouse organs including the heart, liver, spleen, lungs, kidneys, tumors, or healed skin were dissected, fixed with 4% paraformaldehyde solution, and embedded in paraffin. Slices were then sectioned and stained with H&E dyes and finally subjected to microscope observation. The alkaline hematoxylin stained the nucleus with purple-blue, while the acidic eosin stained the cytoplasm with red.

### Statistical Analysis

Statistical significance was determined using one-way analysis of variance (ANOVA). *p* > 0.05, indicated by “ns”, was considered to be not significant. *p* < 0.05, indicated by “*”, was considered to be statistically significant. *p* < 0.01 was indicated by “**”, and *p* < 0.001 was indicated by “***”.

## Supporting Information Available

Materials and instruments, polymer synthesis and characterization, and supplementary figures and tables (PDF)

## References

[ref1] Liu Y. J.; Bhattarai P.; Dai Z. F.; Chen X. Y. Photothermal Therapy and Photoacoustic Imaging via Nanotheranostics in Fighting Cancer. Chem. Soc. Rev. 2019, 48, 2053–2108. 10.1039/C8CS00618K. 30259015 PMC6437026

[ref2] Li S.; Zhang W.; Xing R.; Yuan C.; Xue H.; Yan X. Supramolecular Nanofibrils Formed by Coassembly of Clinically Approved Drugs for Tumor Photothermal Immunotherapy. Adv. Mater. 2021, 33, 2100595 10.1002/adma.202100595. 34288153

[ref3] Xing R.; Zou Q.; Yuan C.; Zhao L.; Chang R.; Yan X. Self-Assembling Endogenous Biliverdin as a Versatile Near-Infrared Photothermal Nanoagent for Cancer Theranostics. Adv. Mater. 2019, 31, 1900822 10.1002/adma.201900822. 30828877

[ref4] Xie L. S.; Li J.; Wang G. H.; Sang W.; Xu M. Z.; Li W. X.; Yan J.; Li B.; Zhang Z.; Zhao Q.; Yuan Z.; Fan Q. L.; Dai Y. L. Phototheranostic Metal-Phenolic Networks with Antiexosomal PDL1 Enhanced Ferroptosis for Synergistic Immunotherapy. J. Am. Chem. Soc. 2022, 144, 787–797. 10.1021/jacs.1c09753. 34985903

[ref5] Li J.; Xie L.; Li B.; Yin C.; Wang G.; Sang W.; Li W.; Tian H.; Zhang Z.; Zhang X.; Fan Q.; Dai Y. Engineering a Hydrogen-Sulfide-Based Nanomodulator to Normalize Hyperactive Photothermal Immunogenicity for Combination Cancer Therapy. Adv. Mater. 2021, 33, 2008481 10.1002/adma.202008481. 33899283

[ref6] Chang R.; Zhao L. Y.; Xing R. R.; Li J. B.; Yan X. H. Functional Chromopeptide Nanoarchitectonics: Molecular Design, Self-Assembly and Biological Applications. Chem. Soc. Rev. 2023, 52, 2688–2712. 10.1039/D2CS00675H. 36987746

[ref7] Zhu Y.; Niu X.; Ding C.; Lin Y.; Fang W.; Yan L.; Cheng J.; Zou J.; Tian Y.; Huang W.; Huang W.; Pan Y.; Wu T.; Chen X.; Kang D. Carrier-Free Self-Assembly Nano-Sonosensitizers for Sonodynamic-Amplified Cuproptosis-Ferroptosis in Glioblastoma Therapy. Adv. Sci. 2024, 11, 2402516 10.1002/advs.202402516. PMC11187904 38582500

[ref8] Zhu Y.; Niu X. G.; Wu T. T.; Cheng J. J.; Zou J. H.; Pan Y. B.; Tian Y.; Huang W.; Ding C. Y.; Lin Y. X.; Kang D. Z.; Chen X. Y. Metal-Phenolic Nanocatalyst Rewires Metabolic Vulnerability for Catalytically Amplified Ferroptosis. Chem. Eng. J. 2024, 485, 150126 10.1016/j.cej.2024.150126.

[ref9] Zhao L.; Liu Y.; Chang R.; Xing R.; Yan X. Supramolecular Photothermal Nanomaterials as an Emerging Paradigm toward Precision Cancer Therapy. Adv. Funct. Mater. 2019, 29, 1806877 10.1002/adfm.201806877.

[ref10] Shi J. J.; Kantoff P. W.; Wooster R.; Farokhzad O. C. Cancer Nanomedicine: Progress, Challenges and Opportunities. Nat. Rev. Cancer 2017, 17, 20–37. 10.1038/nrc.2016.108. 27834398 PMC5575742

[ref11] Lenders V.; Escudero R.; Koutsoumpou X.; Armengol Álvarez L.; Rozenski J.; Soenen S. J.; Zhao Z.; Mitragotri S.; Baatsen P.; Allegaert K.; Toelen J.; Manshian B. B.; et al. Modularity of RBC Hitchhiking with Polymeric Nanoparticles: Testing the Limits of Non-Covalent Adsorption. J. Nanobiotechnol. 2022, 20, 333 10.1186/s12951-022-01544-0. PMC9287723 35842697

[ref12] Glassman P. M.; Hood E. D.; Ferguson L. T.; Zhao Z.; Siegel D. L.; Mitragotri S.; Brenner J. S.; Muzykantov V. R. Red Blood Cells: The Metamorphosis of a Neglected Carrier Into the Natural Mothership for Artificial Nanocarriers. Adv. Drug Delivery Rev. 2021, 178, 113992 10.1016/j.addr.2021.113992. PMC8556370 34597748

[ref13] Sun T. M.; Zhang Y. S.; Pang B.; Hyun D. C.; Yang M. X.; Xia Y. N. Engineered Nanoparticles for Drug Delivery in Cancer Therapy. Angew. Chem., Int. Ed. 2014, 53, 12320–12364. 10.1002/anie.201403036. 25294565

[ref14] Chauhan V. P.; Popović Z.; Chen O.; Cui J.; Fukumura D.; Bawendi M. G.; Jain R. K. Fluorescent Nanorods and Nanospheres for Real-Time In Vivo Probing of Nanoparticle Shape-Dependent Tumor Penetration. Angew. Chem., Int. Ed. 2011, 50, 11417–11420. 10.1002/anie.201104449. PMC3260125 22113800

[ref15] Lim E. K.; Kim T.; Paik S.; Haam S.; Huh Y. M.; Lee K. Nanomaterials for Theranostics: Recent Advances and Future Challenges. Chem. Rev. 2015, 115, 327–394. 10.1021/cr300213b. 25423180

[ref16] Abdelmohsen L. K. E. A.; Williams D. S.; Pille J.; Ozel S. G.; Rikken R. S. M.; Wilson D. A.; van Hest J. C. M. Formation of Well-Defined, Functional Nanotubes via Osmotically Induced Shape Transformation of Biodegradable Polymersomes. J. Am. Chem. Soc. 2016, 138, 9353–9356. 10.1021/jacs.6b03984. 27374777 PMC4974604

[ref17] Pijpers I. A. B.; Abdelmohsen L. K. E. A.; Williams D. S.; van Hest J. C. M. Morphology Under Control: Engineering Biodegradable Stomatocytes. ACS Macro Lett. 2017, 6, 1217–1222. 10.1021/acsmacrolett.7b00723. 29214115 PMC5708263

[ref18] Shao J. X.; Cao S. P.; Williams D. S.; Abdelmohsen L. K. E. A.; van Hest J. C. M. Photoactivated Polymersome Nanomotors: Traversing Biological Barriers. Angew. Chem., Int. Ed. 2020, 59, 16918–16925. 10.1002/anie.202003748. PMC7540338 32533754

[ref19] Scheerstra J. F.; Wauters A. C.; Tel J.; Abdelmohsen L. K. E. A.; van Hest J. C. M. Polymersomes As a Potential Platform for Cancer Immunotherapy. Mater. Today Adv. 2022, 13, 100203 10.1016/j.mtadv.2021.100203.

[ref20] Shao J.; Pijpers I. A. B.; Cao S.; Williams D. S.; Yan X.; Li J.; Abdelmohsen L. K. E. A.; van Hest J. C. M. Biomorphic Engineering of Multifunctional Polylactide Stomatocytes toward Therapeutic Nano-Red Blood Cells. Adv. Sci. 2019, 6, 1801678 10.1002/advs.201801678. PMC6402394 30886797

[ref21] Kapate N.; Clegg J. R.; Mitragotri S. Non-Spherical Micro- and Nanoparticles for Drug Delivery: Progress Over 15 Years. Adv. Drug Delivery Rev. 2021, 177, 113807 10.1016/j.addr.2021.05.017. 34023331

[ref22] Wauters A. C.; Pijpers I. A. B.; Mason A. F.; Williams D. S.; Tel J.; Abdelmohsen L. K. E. A.; van Hest J. C. M. Development of Morphologically Discrete PEG–PDLLA Nanotubes for Precision Nanomedicine. Biomacromolecules 2019, 20, 177–183. 10.1021/acs.biomac.8b01245. 30265794 PMC6335608

[ref23] Varma L. T.; Singh N.; Gorain B.; Choudhury H.; Tambuwala M. M.; Kesharwani P.; Shukla R. Recent Advances in Self-Assembled Nanoparticles for Drug Delivery. Curr. Drug Delivery 2020, 17, 279–291. 10.2174/1567201817666200210122340. 32039683

[ref24] Mujtaba M.; Negi A.; King A. W. T.; Zare M.; Kuncova-Kallio J. Surface Modifications of Nanocellulose for Drug Delivery Applications; a Critical Review. Curr. Opin. Biomed. Eng. 2023, 28, 100475 10.1016/j.cobme.2023.100475.

[ref25] Sakamoto K.; Ohno-Okumura E. Syntheses and Functional Properties of Phthalocyanines. Materials 2009, 2, 1127–1179. 10.3390/ma2031127.

[ref26] Kasha M.; Rawls H. R.; Ashraf El-Bayoumi M. The Exciton Model in Molecular Spectroscopy. Pure Appl. Chem. 1965, 11, 371–392. 10.1351/pac196511030371.

[ref27] Su M. H.; Han Q. J.; Yan X. S.; Liu Y. N.; Luo P.; Zhai W. H.; Zhang Q. Z.; Li L. Y.; Li C. H. A Supramolecular Strategy to Engineering a Non-photobleaching and Near-Infrared Absorbing Nano-J-Aggregate for Efficient Photothermal Therapy. ACS Nano 2021, 15, 5032–5042. 10.1021/acsnano.0c09993. 33635051

[ref28] Chang R.; Zou Q.; Zhao L.; Liu Y.; Xing R.; Yan X. Amino-Acid-Encoded Supramolecular Photothermal Nanomedicine for Enhanced Cancer Therapy. Adv. Mater. 2022, 34, 2200139 10.1002/adma.202200139. 35178775

[ref29] Zhao L. Y.; Liu Y. M.; Xing R. R.; Yan X. H. Supramolecular Photothermal Effects: A Promising Mechanism for Efficient Thermal Conversion. Angew. Chem., Int. Ed. 2020, 59, 3793–3801. 10.1002/anie.201909825. 31571353

[ref30] Fu M.; Yang Y.; Zhang Z.; He Y.; Wang Y.; Liu C.; Xu X.; Lin J.; Yan F. Biosynthesis of Melanin Nanoparticles for Photoacoustic Imaging Guided Photothermal Therapy. Small 2023, 19, 2205343 10.1002/smll.202205343. 36581563

[ref31] Wang L. H. V.; Hu S. Photoacoustic Tomography: In Vivo Imaging from Organelles to Organs. Science 2012, 335, 1458–1462. 10.1126/science.1216210. 22442475 PMC3322413

[ref32] Manson J.; Kumar D.; Meenan B. J.; Dixon D. Polyethylene glycol functionalized gold nanoparticles: the influence of capping density on stability in various media. Gold Bull. 2011, 44, 99–105. 10.1007/s13404-011-0015-8.

[ref33] Matsumura Y.; Maeda H. A New Concept for Macromolecular Therapeutics in Cancer Chemotherapy: Mechanism of Tumoritropic Accumulation of Proteins and the Antitumor Agent Smancs. Cancer Res. 1986, 46, 6387–6392. 2946403

[ref34] Cheng D. B.; Yang P. P.; Cong Y.; Liu F. H.; Qiao Z. Y.; Wang H. One-Pot Synthesis of pH-Responsive Hyperbranched Polymer–Peptide Conjugates with Enhanced Stability and Loading Efficiency for Combined Cancer Therapy. Polym. Chem. 2017, 8, 2462–2471. 10.1039/C7PY00101K.

[ref35] Hu C. M. J.; Fang R. H.; Luk B. T.; Chen K. N. H.; Carpenter C.; Gao W. W.; Zhang K.; Zhang L. F. ‘Marker-of-Self’ Functionalization of Nanoscale Particles Through a Top-Down Cellular Membrane Coating Approach. Nanoscale 2013, 5, 2664–2668. 10.1039/c3nr00015j. 23462967 PMC3667603

