# Supplementary material for: Flexible Morphological Regulation of Photothermal Nanodrugs: Understanding the Relationship between the Structure, Photothermal Effect, and Tumoral Biodistribution
Source: ACS Nano. 2025 Jan 10;19(2):2799–808. doi: 10.1021/acsnano.4c15587 (PMC11760176; doi:10.1021/acsnano.4c15587)
Supplement: Supplementary file 1 — nn4c15587_si_001.pdf [file nn4c15587_si_001.pdf]

Supporting Information for

Flexible morphological regulation of photothermal nanodrugs: understanding the relationship of structure, photothermal effect, and tumoral biodistribution

Shukun Li,[1,2,‡] *Yudong Li,*[1,‡] *Guizhi Shen,*[2] *Juping Sun,*[2,3] *Loai K. E. A. Abdelmohsen,*[1] *Xuehai Yan,*[2,3,*] *and Jan C. M. van Hest*[1,*]

[1] Bio-Organic Chemistry, Institute for Complex Molecular Systems, Eindhoven University of Technology, P.O. Box 513, 5600 MB Eindhoven, The Netherlands

[2] State Key Laboratory of Biochemical Engineering, Institute of Process Engineering, Beijing 100190, China

[3] School of Chemical Engineering, University of Chinese Academy of Sciences, Beijing 100049, China.

[‡] These authors contributed equally.

CONTENTS

SUPPLEMENTARY MATERIALS

SUPPLEMENTARY INSTRUMENTS

SYNTHESIS AND CHARACTERIZATION OF PEG–PLA BLOCK COPOLYMERS

SUPPLEMENTARY FIGURES 1-9

SUPPLEMENTARY TABLES 1-6

REFERENCE

SUPPLEMENTARY MATERIALS

All materials were used as received without further purification unless otherwise indicated. α-Methoxy-ω-hydroxy poly(ethylene glycol) (mPEG, average M_n 1 kDa and 2 kDa) was purchased from Biopharma PEG Scientific (Biochempeg Scientific) and Rapp Polymere GmbH, respectively. Dichloromethane (DCM), tetrahydrofuran (THF), and dioxane were obtained from Biosolve Chimie. 3,6-dimethyl-1,4-dioxane-2,5-dione (D,L-lactide, 99%), 1,8-diazabicyclo[5.4.0]undec-7-ene (DBU, 98%), cobalt phthalocyanine (Pc), sodium chloride, calcein AM, propidium iodide (PI), paraformaldehyde and all other chemicals were supplied by Sigma-Aldrich unless otherwise stated. Dialysis membranes (MWCO: 12–14 kDa, Spectra/Pro®) were used for nanoparticle purification. Water used in this work was ultrapure Milli-Q (Millipore) water (18.2 MΩ·cm). Cell culture Dulbecco's modified Eagle medium (DMEM), heat-inactivated fetal bovine serum (FBS), Dulbecco's phosphate-buffered saline (PBS), trypsin-EDTA, and penicillin-streptomycin were purchased from BioLegend Co. Human breast cancer cells (MCF-7) were provided by the Chinese Academy of Medical Sciences. Female BALB/c mice were provided by Beijing HFK Bioscience Co., Ltd.

SUPPLEMENTARY INSTRUMENTS

Nuclear Magnetic Resonance (NMR) Spectroscopy. Proton nuclear magnetic resonance (^{1}H-NMR) measurements were conducted on a Bruker Avance 400 MHz Ultrashield spectrometer equipped with a Bruker SampleCase autosampler, using $CDCl_3$ as the solvent and TMS as the internal standard. The obtained spectra were analyzed using MestReNova NMR analysis software.

Gel Permeation Chromatography (GPC). The molecular weights and dispersity (*Đ*) of the copolymers were characterized by using a Prominence-I GPC system (Shimadzu) equipped with a PL gel 5 μm mixed D (Polymer Laboratories) and differential refractive index (RI) and ultraviolet (UV) detectors. THF was used as the eluent with a flow rate of 1 mL min^{-1}. Polystyrene standards (Polymer Laboratories) were used for calibration. GPC chromatograms were obtained from the RI detector unless otherwise indicated.

Dynamic Light Scattering (DLS) Measurements. The hydrodynamic diameter (D_h) and polydispersity index (PDI) of the nanodrugs were measured using a Malvern Instruments Zetasizer (model Nano ZSP) equipped with a 633 nm He-Ne laser and an avalanche photodiode detector at 25 °C. Type ZEN0040 (100 μL sample volume) disposable cuvettes were used for DLS measurements. Zetasizer software was used to process and analyze the data.

Ultraviolet–Visible (UV–Vis) Spectroscopy. UV–Vis spectra were recorded with a Cary 3500 Multicell UV–Vis spectrophotometer (Agilent Technologies) using a quartz cuvette (1 mL sample volume).

Fluorescence Spectroscopy. Fluorescence intensity measurements were performed on a Cary Eclipse fluorescence spectrometer (Agilent Technologies) using a quartz cuvette (1 mL sample volume).

Cryogenic Transmission Electron Microscopy (cryo-TEM). Cryo-TEM experiments were performed on the TU/e CryoTitan (Thermo Fisher Scientific) equipped with a field emission gun and autoloader and operated at 300 kV acceleration voltage in low-dose bright-field TEM mode. Samples for cryo-TEM were prepared by glow-discharging the grids (Quantifoil Cu grid with R 2/2 holey carbon films, Quantifoil Micro Tools GmbH, part of the SPT Life Sciences group) in a Cressington 208 carbon coater for 40 seconds. Then, 3 μL of sample (ca. 2.3 mg/mL) was pipetted on the grid and blotted in a Vitrobot MARK IV at room temperature and 100% humidity. The grid was blotted for 3 seconds (offset −3) and directly plunged and vitrified in liquid ethane. Cryo-TEM images were acquired in zero loss energy filtering mode (Gatan GIF 2002, 20 eV energy slit) with a CCD camera (Gatan model 794). Processing of all electron microscope images was performed with Fiji 2.7.0 software (ImageJ2).

pH Meter. FiveEasy Plus FP20 pH Meter (Mettler Toledo) was used to monitor the pH.

Freeze-Dryer. Lyophilization was performed on an Alpha 2-4 LSCbasic freeze-dryer (Christ).

Centrifuge. Centrifugation was carried out on an Eppendorf 5424R microcentrifuge or a VWR Mega Star 600 centrifuge.

660 nm Laser. The 660 nm laser produced by Changchun Laser Optoelectronics Technology Co., Ltd and with a power density range of 1-5000 mW was used to irradiate nanodrugs or tumor tissues.

Thermo-Detector. The temperature was recorded by a UNI UT322 thermo-detector.

Confocal Laser Scanning Microscopy (CLSM). The CLSM images were obtained by the Leica SP5 CLSM.

Thermal Imaging Camera. The FLIR E5 thermal image camera was used to record the temperature of tumor tissues *in vivo*.

Photoacoustic (PA) Imaging System. The VisualSonics Vevo® LAZR-X PA imaging system was used to record the PA signal of tumor tissues *in vivo*.

SYNTHESIS AND CHARACTERIZATION OF PEG–PLA BLOCK COPOLYMERS

The synthesis of PEG-*b*-PLA was performed according to a previously reported procedure with several modifications[1]. To synthesize copolymer with composition of PEG_{22}-*b*-PLA_{45}, 97 mg (0.1 mmol) of $mPEG_{22}$ and 650 mg (4.5 mmol, 45 equiv.) of D,L-lactide were first weighed in a 100-mL round-bottom flask. Subsequently, dry toluene (ca. 50 mL) was added to the flask and the solvent was evaporated to dry the contents before polymerization. The dried reagents were then re-dissolved in dry DCM ([monomer] = 0.5 M) and DBU was added (0.5 equiv. with respect to [initiator]; 0.05 mmol) under argon. The reaction proceeded with magnetic stirring at room temperature. The progress of the reaction was monitored by ^{1}H-NMR spectroscopy until the monomer conversion exceeded 96%. In a typical reaction, it reached this point within 2 h. Subsequently, the reaction mixture was diluted with DCM and washed twice with 1M $KHSO_4$ and once with brine. The organic solution was dried using Na_2SO_4. After evaporating most of the solvent, the concentrated copolymer solution was precipitated into ice-cold diethyl ether. The resulting waxy solid was partially dried under argon, dissolved in dioxane, and lyophilized to yield a white powder (yield = 70-80%). Similarly, PEG_{44}-*b*-PLA_{90} and PEG_{44}-*b*-PLA_{120} were synthesized.

The synthesized copolymers were characterized using ^{1}H NMR spectroscopy and GPC to determine copolymer composition and polydispersity, respectively. ^{1}H-NMR ($CDCl_3$, 400 MHz, δ in ppm): 5.28-5.10 ppm (m, PLA C**H**), 3.64 ppm (s, PEG backbone C$\mathbf{H_2}$), 3.38 ppm (s, PEG C$\mathbf{H_3}$O), 1.65-1.45 ppm (m, PLA C$\mathbf{H_3}$).

SUPPLEMENTARY FIGURES

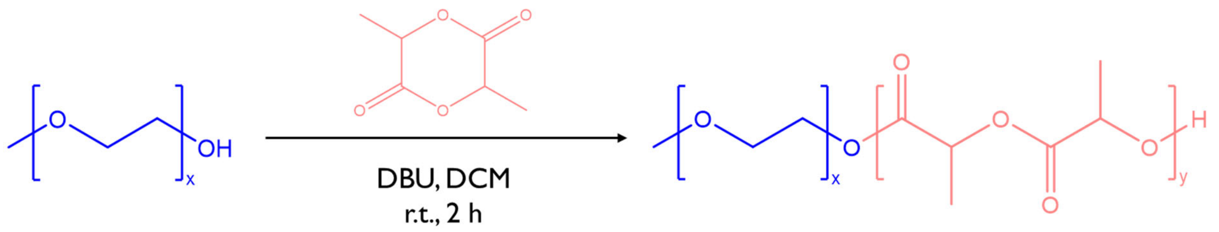

Figure S1. Scheme showing the synthetic route toward PEG_x-*b*-PLA_y block copolymers *via* ring-opening polymerization in dry DCM, with x/y = 22/45, 44/90, or 44/120.

a

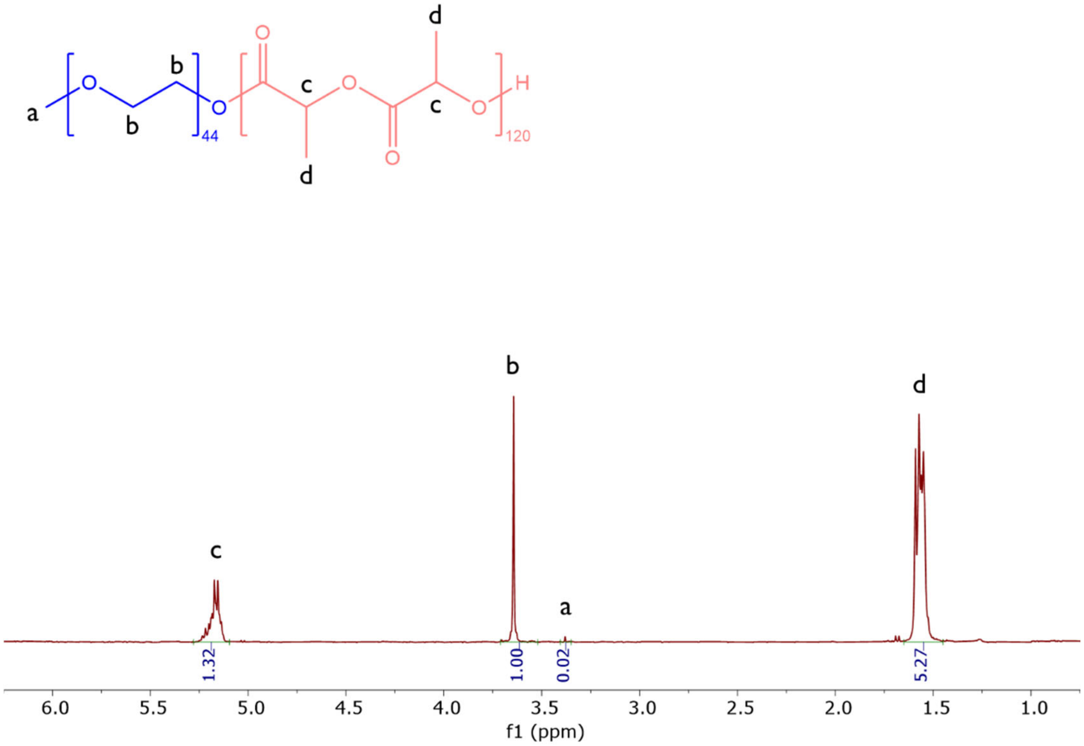

a
b
c
d
44
120
O
H
1.32
1.00
0.02
5.27
6.0 5.5 5.0 4.5 4.0 3.5 3.0 2.5 2.0 1.5 1.0
f1 (ppm)

b

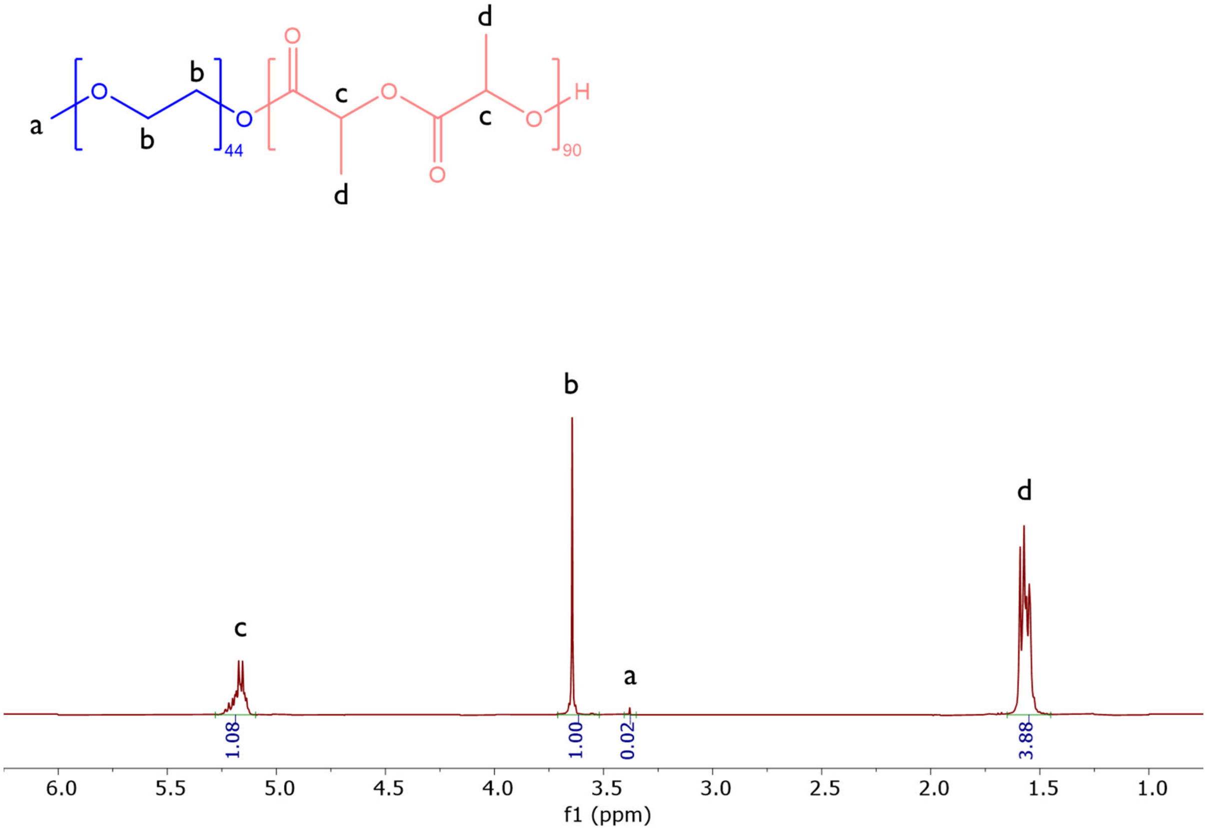

a
b
c
d
44
90
O
H
1.08
1.00
0.02
3.88
6.0 5.5 5.0 4.5 4.0 3.5 3.0 2.5 2.0 1.5 1.0
f1 (ppm)

c

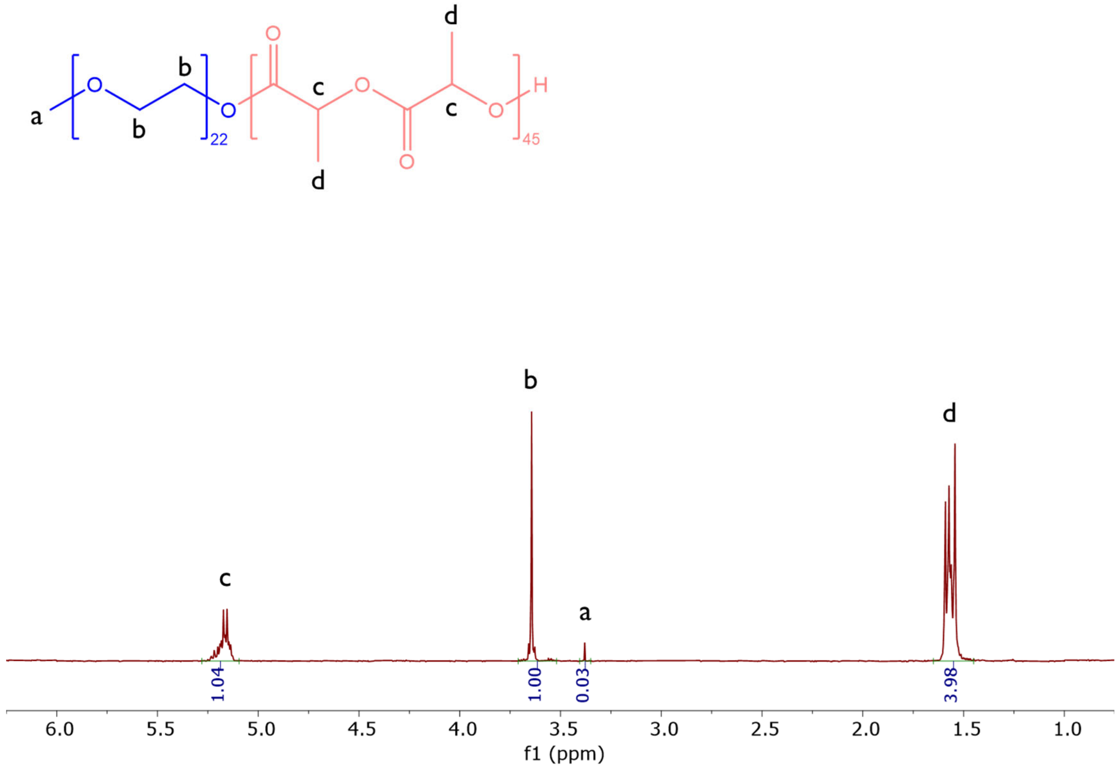

Figure S2. ^{1}H NMR spectra of **a**) PEG$_{44}$-*b*-PLA$_{120}$, **b**) PEG$_{44}$-*b*-PLA$_{90}$, and **c**) PEG$_{22}$-*b*-PLA$_{45}$.

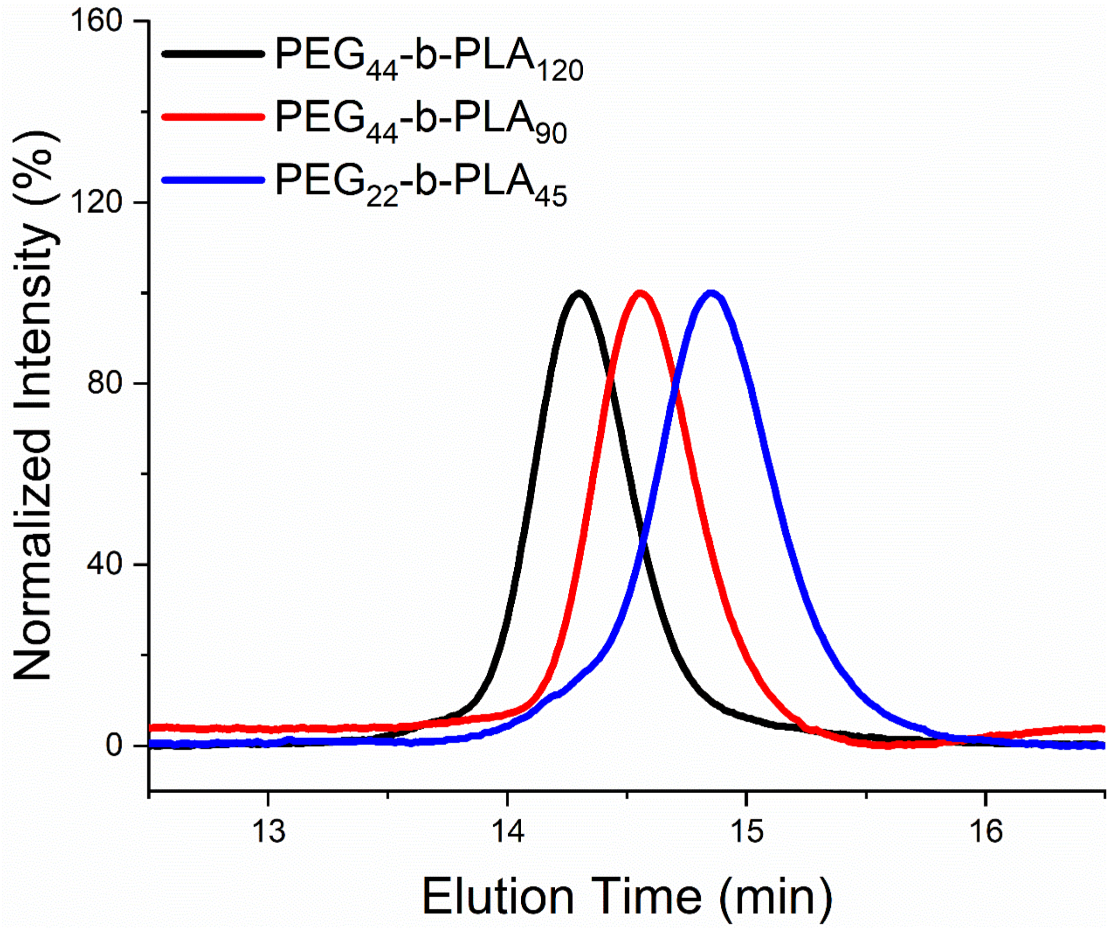

Figure S3. GPC traces of PEG_{22}-*b*-PLA_{45}, PEG_{44}-*b*-PLA_{90}, and PEG_{44}-*b*-PLA_{120}. THF was used as the eluent and polystyrene standards were used for calibration.

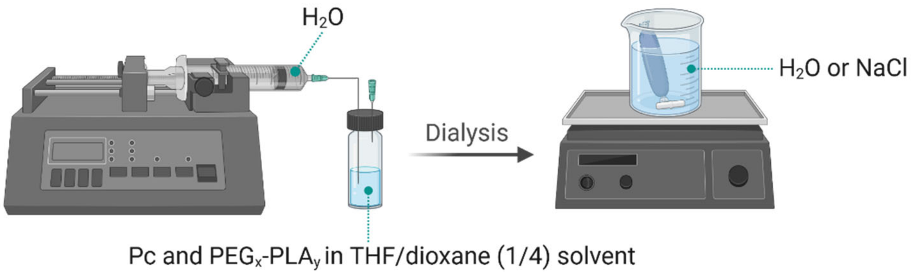

Figure S4. Preparation scheme of co-assembly of PEG_x-*b*-PLA_y polymers and phthalocyanine (Pc).

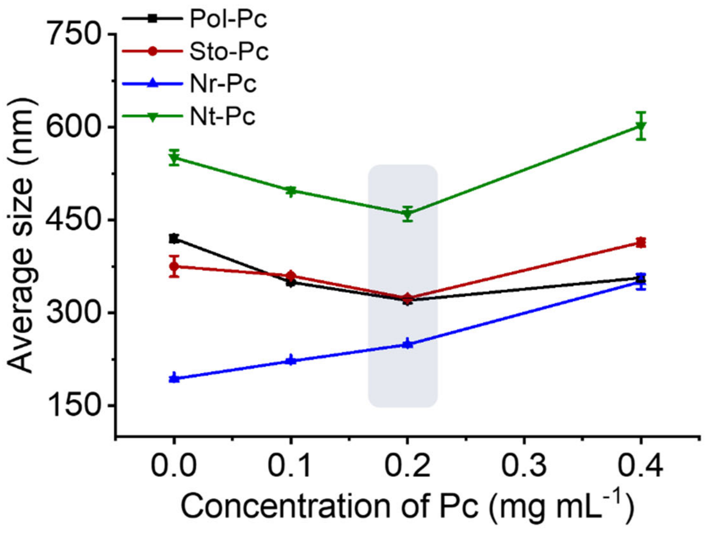

Figure S5. Mass ratio optimization of co-assembly preparation of polymers and Pc.

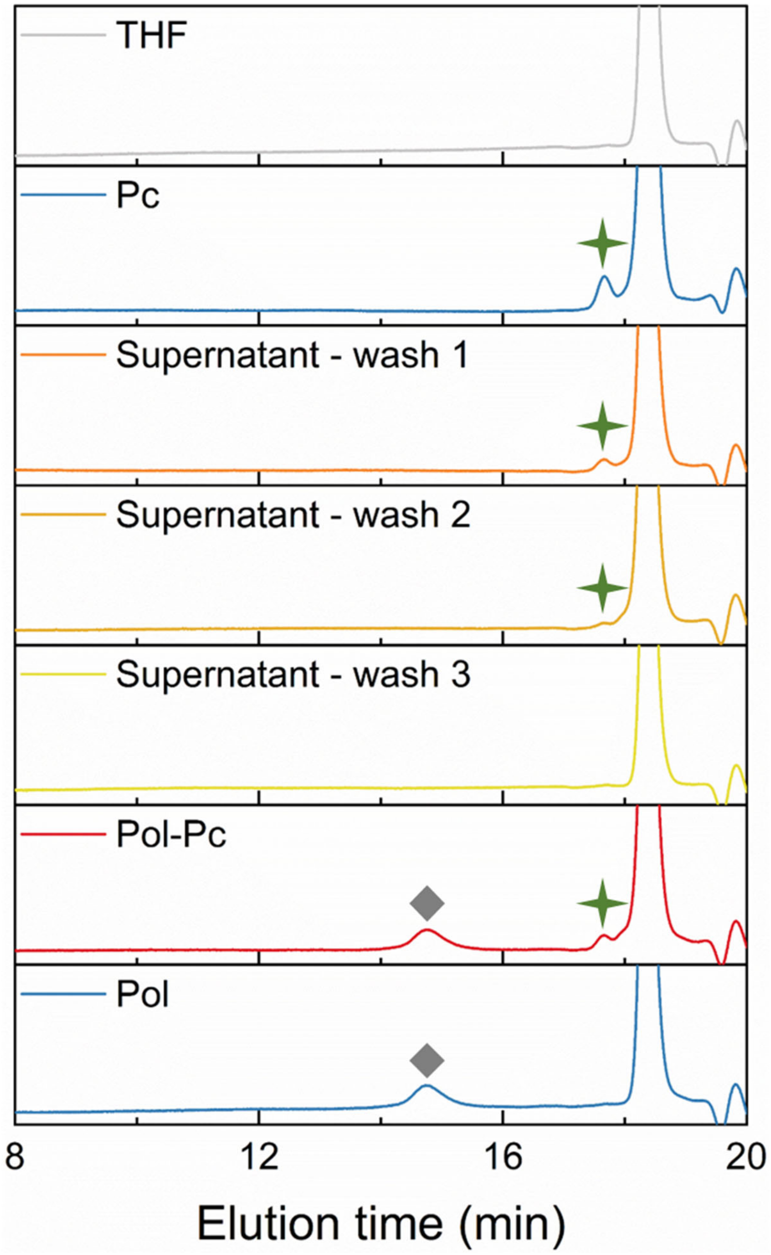

Figure S6. Chromatography measurements showing successful encapsulation of Pc in Pol. THF was used as the eluent. Gray diamond ◆: polymer peak; green star ✦: Pc.

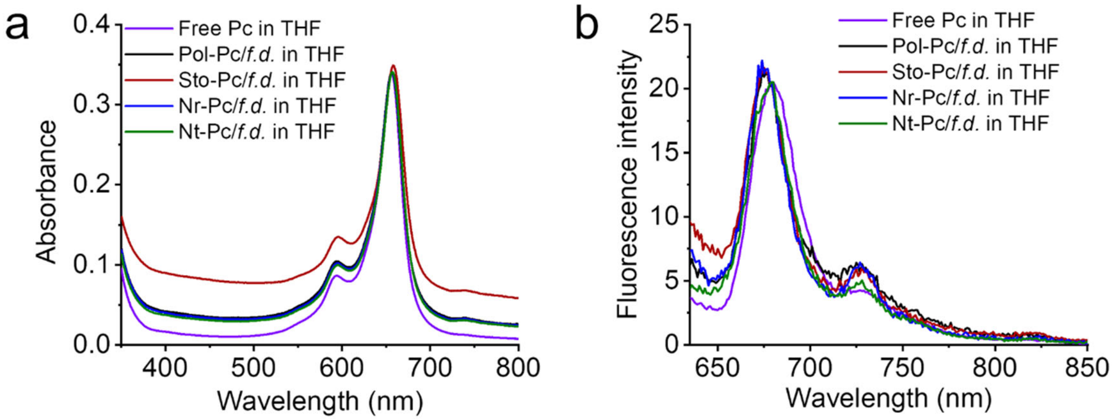

Figure S7. Spectra of nanodrugs. (a) Absorption spectra and (b) fluorescence spectra of nanodrugs after freeze drying and dissolving in THF. The concentration of Pc used in these figures is 0.01 mg mL^{-1}. Freeze-dried is abbreviated as *f.d.*.

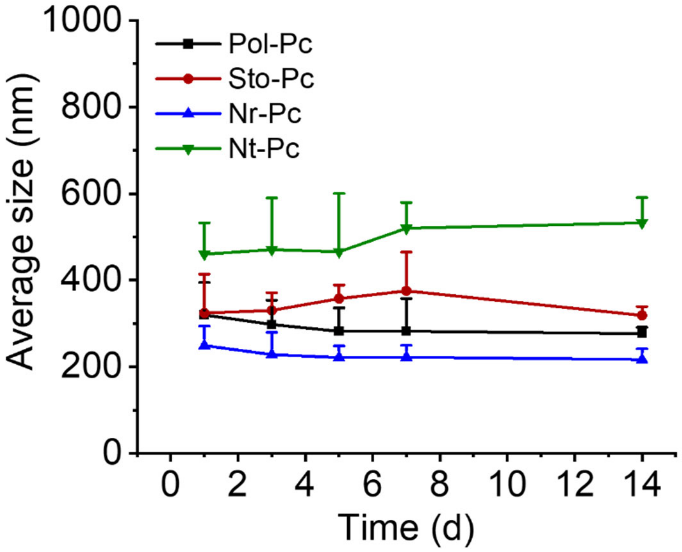

Figure S8. Average size change of four kinds of nanodrugs during storage.

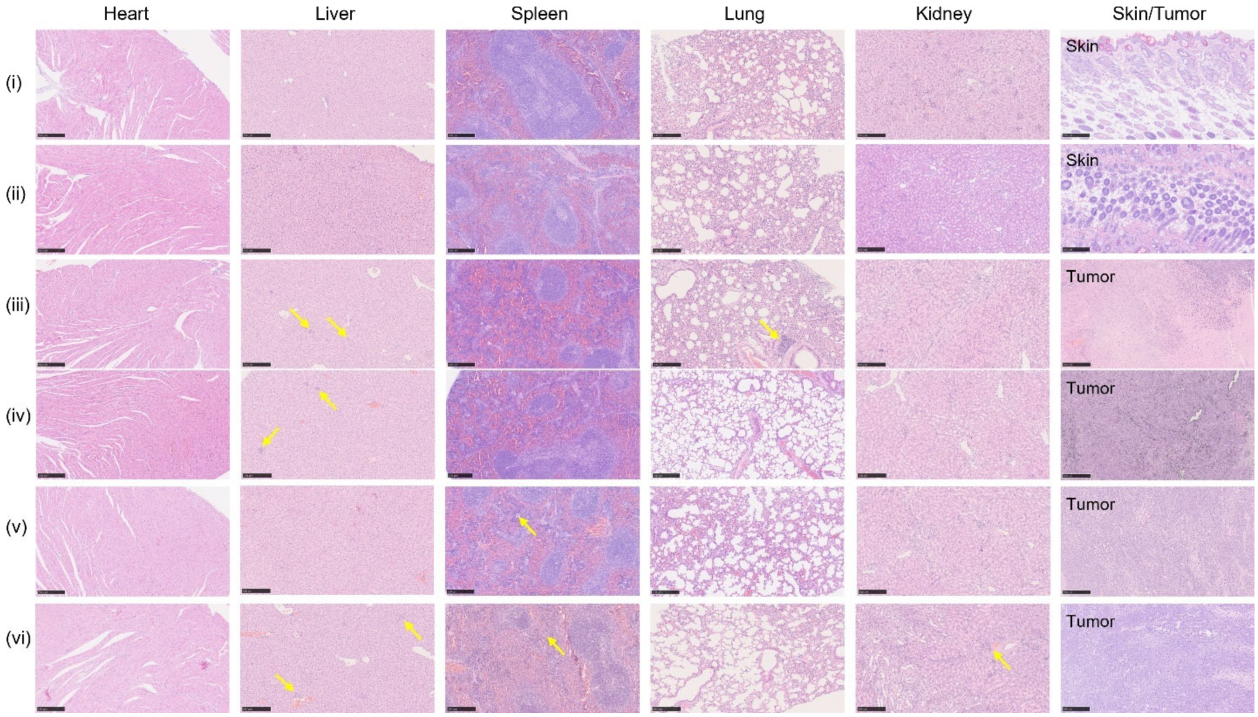

Figure S9. H&E images of mice heart, liver, spleen, lung, kidney, tumor or skin. The yellow arrows indicate tumor lesions. The groups are (i) Nr-Pc + laser, (ii) Sto-Pc + laser, (iii) Control + laser, (iv) Nr-Pc, (v) Sto-Pc, and (vi) Control.

SUPPLEMENTARY TABLES

Table S1. Summary of PEG-*b*-PLA block copolymers used for nanoparticle preparation in this study.

Polymer	Composition by ^{1}H NMR	$Đ$ from GPC
PEG_{44}-*b*-PLA_{120}	PEG_{44}-*b*-PLA_{116}	1.07
PEG_{44}-*b*-PLA_{90}	PEG_{44}-*b*-PLA_{95}	1.05
PEG_{22}-*b*-PLA_{45}	PEG_{22}-*b*-PLA_{46}	1.09

Table S2. Preparation parameters of nanodrugs.

Morphology	Molecule	Polymer (mg mL^{-1})	Pc (mg mL^{-1})	Dialysis
Polymersomes (Pol)	PEG_{44}-*b*-PLA_{120}	5	0, 0.1, 0.2, 0.4	H_2O
Stomatocytes (Sto)	PEG_{44}-*b*-PLA_{120}			75 mM NaCl
Nanorods (Nr)	PEG_{44}-*b*-PLA_{90}			H_2O
Nanotubes (Nt)	PEG_{22}-*b*-PLA_{45}			50 mM NaCl

Table S3. Average hydrodynamic diameter (D_h) and PDI of the nanodrugs that are prepared at different mass ratios of polymer to phthalocyanine.

Pc	Pol-Pc		Sto-Pc		Nr-Pc		Nt-Pc	
(mg mL^{-1})	D_h (nm)	PDI	D_h (nm)	PDI	D_h (nm)	PDI	D_h (nm)	PDI
0	420.1	0.171	375.3	0.120	153.5	0.101	550.9	0.247
0.1	349.8	0.171	360.0	0.194	176.1	0.257	498.1	0.202
0.2	320.1	0.161	323.8	0.084	248.9	0.336	460.1	0.118
0.4	356.8	0.165	413.9	0.183	350.5	0.405	602.3	0.411

Table S4. Changes of Pc Q band, shift and tentative Pc organization in nanodrugs.

Group	Q band (nm)		Shift	Tentative Pc organization
	593	655	Ref.	/
Pol-Pc	607	674	Bathochromic	J-aggregation
Sto-Pc	601	669	Bathochromic	J-aggregation
Nr-Pc	612	686	Bathochromic	J-aggregation
Nt-Pc	610	681	Bathochromic	J-aggregation

Table S5. Encapsulation efficiency (EE) of Pc molecules in nanodrugs

Group	EE (%)
Pol-Pc	26
Sto-Pc	19
Nr-Pc	39
Nt-Pc	11

Table S6. Photothermal parameters summary of nanodrugs

Group	Photothermal conversion	
	ΔT (T_{max}-T_{sur})	η (%)
Pol-Pc	17.1	42.2
Sto-Pc	17.5	41.6
Nr-Pc	20.7	45.7
Nt-Pc	15.3	43.3
Water (control)	2.3	/

REFERENCE

1. Abdelmohsen, L. K.; Williams, D. S.; Pille, J.; Ozel, S. G.; Rikken, R. S.; Wilson, D. A.; van Hest, J. C., Formation of well-defined, functional nanotubes via osmotically induced shape transformation of biodegradable polymersomes. *J. Am. Chem. Soc.* **2016**, *138*, 9353-9356.
